# Patient preferences for the provision of NHS medicines helpline services: a discrete choice experiment

**DOI:** 10.1080/20523211.2024.2404973

**Published:** 2024-10-01

**Authors:** Ben Ashby, Matthew D. Jones

**Affiliations:** a Institute for Mathematical Innovation, University of Bath, Bath, UK; b Department of Life Sciences, University of Bath, Bath, UK

**Keywords:** Discrete choice experiment, drug information, hospital discharge, medicines helpline, medicines information, patient discharge, patient preferences

## Abstract

**Introduction:**

Patient medicines helpline services (PMHS) can reduce harm and improve medicines adherence and patient satisfaction after hospital discharge. There is little evidence of which PMHS attributes are most important to patients. This would enable PMHS providers to prioritise their limited resources to maximise patient benefit.

**Methods:**

Patient preferences for PMHS attributes were measured using a discrete choice experiment. Seven attributes were identified from past research, documentary analysis and stakeholder consultation. These were used to produce a D-efficient design with two blocks of ten choice sets incorporated into an online survey. Adults in the UK who took more than one medicine were eligible to complete the survey and were recruited via the Research for the Future database. Preferences were estimated using conditional logistic regression. Associations between participant characteristics and preferences were investigated with latent class models.

**Results:**

460 participants completed the survey. The most valued attributes were weekend opening (willingness-to-pay, WTP: £11.20), evening opening (WTP: £8.89), and receiving an answer on the same day (WTP: £9.27). Alternative contact methods, immediate contact with a pharmacist and helpline location were valued less. Female gender and full-time work were associated with variation in preferences. For one latent class containing 27% of participants, PMHS location at the patient’s hospital was the most valued attribute.

**Discussion:**

PMHS providers should prioritise extended opening hours and answering questions on the same day. Limitations include a non-representative sample in terms of ethnicity, education and geography, and the exclusion of people without internet access.

## Introduction

It is estimated that over 3 million medication errors occur during hospital discharge in England each year, with approximately 20% of discharge prescriptions associated with an error (Elliott et al., 2021). In addition, international studies have found that many patients (ranging from 12% to 93%) experience medication-related problems following hospital discharge, including lack of efficacy, adverse effects, drug interactions, difficulty using a new regimen and lack of information (Ellitt et al., 2010; Forster et al., 2003; Mackridge et al., 2018; Marvin et al., 2012). To mitigate such problems, many hospitals have established a patient medicines helpline service (PMHS), which enables discharged patients to contact a pharmacy professional to address their questions and concerns. These services, which can be considered a form of telehealth, are particularly common in the United Kingdom (UK), where a 2017 survey found that they were provided by 52% of National Health Service (NHS) trusts in England (Williams et al., 2018), but they have also been established in many other countries including Canada, France, Germany, Israel, the Netherlands, Saudi Arabia and the United States of America (Williams et al., 2019b). A recent systematic review found evidence suggesting that PMHS reduce patient harm and visits to other healthcare providers (e.g. GPs) while improving medicines adherence and patient satisfaction, although this was of moderate quality and high risk of bias (Williams et al., 2019b).

PMHS in England received a median of five enquiries per week in 2017 (Williams et al., 2018). There were 8,683,046 discharges (European Union, 2022) from 1,920 hospitals (Organisation for Economic Co-operation and Development, 2023) in the UK in 2017, an average of 87 discharges per hospital per week. If a conservative 30% of these patients experienced a medication-related problem (Ellitt et al., 2010; Forster et al., 2003; Mackridge et al., 2018; Marvin et al., 2012), 26 people per hospital per week might be expected to contact a PMHS. This suggests that PMHS are significantly underused, limiting the number of patients who might benefit. Many factors contribute to such underuse, but one is likely to be limited compliance with national PMHS standards (Wills, 2014). In 2017, the ‘satisfactory’ access, availability and promotion standards were fully met by only 54%, 86% and 3% of NHS trusts, respectively (Williams et al., 2018). The equivalent ‘commendable’ standards were met by only 26%, 5% and 40% of NHS trusts. This suggests that many patients may be unaware of, or unable to contact, a PMHS, especially as enquirers in a recent qualitative interview study suggested that these services could be improved by extending their opening hours (Williams et al., 2020b). However, limited compliance with national PMHS standards is attributed to limited resources (Williams et al., 2018), which pharmacy professionals report negatively impact their ability to fully implement PMHS (Williams et al., 2020a). Pharmacy professionals may therefore need to decide which of the national standards to prioritise when developing their PMHS, but there is very limited evidence of which attributes of a PMHS are most important. The only published study examined the importance of a number of attributes reported by 75 previous enquirers of just one PMHS (O'Grady et al., 2021). However, as the participants in this study had already contacted the service, its findings are not representative of people who were unaware of or unable to contact it. Additional research to quantify potential enquirers’ preferences for the way a PMHS is provided is therefore important, as it will enable service providers to prioritise implementation of those standards that are likely to enable the most enquirers to use the service and thus maximise patient benefit.

Discrete choice experiments (DCEs) are a stated preferences method that is widely used for measuring how individuals value different attributes of a product or service (Bridges et al., 2011). They measure how people trade-off one attribute against another, so are valuable for resource prioritisation decisions and are thus an appropriate method for the investigation of PMHS. DCE data are typically collected via a survey that asks participants to choose their preferred service configuration from each of a series of pairs of possible configurations. Each service configuration is described using a set of attributes and levels, where attributes are a characteristic of the way the service is operated (e.g. PMHS opening hours per day) and levels describe the different values that each attribute might take (e.g. opening for 4, 8 or 12 hours/day). It is assumed that participants select the services that would provide them with maximum utility (benefit), so the relative value of each attribute level can be calculated by regression analysis.

This study therefore aimed to measure patient preferences for different attributes of the provision of PMHS using a DCE. We also sought to identify the extent to which personal characteristics are associated with individuals’ preferences for the provision of PMHS services. Reporting is in accordance with the Conjoint Analysis Applications in Health Checklist (Supplemental Material, Appendix 1) (Bridges et al., 2011).

## Materials and methods

### Attributes and levels

DCE attributes must be relevant to the research aims and policy context (Bridges et al., 2011). We therefore pre-specified the following criteria for potential attributes: they must have the potential to affect a patient’s decision to contact a PMHS and be within the control of hospitals providing these services as they decide how to prioritise their resources. Such attributes would therefore either be clear from PMHS advertising or be relevant to patients’ PMHS experience, as this might influence their decision to re-use the service.

We have recently completed a multi-method programme of PMHS research, which included systematic reviews (Williams et al., 2019a; 2019b), a national survey of current PMHS provision (Williams et al., 2018) and qualitative interviews with enquirers (Williams et al., 2020b) and staff (Williams et al., 2020a, 2021). Therefore, additional qualitative research to identify attributes was not necessary. Based on this previous research, an unpublished student pilot study and UK national standards for PMHS (Wills, 2014), the research team identified 34 potential attributes that met the above pre-specified criteria. From these, we prioritised the six attributes most relevant to the research aims, recording reasons for including or excluding each potential attribute (Supplemental Material, Appendix 2). We identified potential levels for the six prioritised attributes. We included the cost of an enquiry as a seventh attribute to allow calculation of participants’ preferences as willingness-to-pay. As enquirers do not pay to use PMHS, this was framed as ‘cost to the NHS’, in line with similar studies of NHS services (Fletcher et al., 2019). We shared this information (Supplemental Material, Appendix 2) with six pharmacists who currently operated a PMHS for feedback on attribute identification, prioritisation, descriptions and levels. These pharmacists’ confirmed we had selected appropriate attributes, but led us to revise some attribute descriptions and include time of day in the levels for opening hours. The final selection of attributes and levels are shown in Table 1.

**Table 1.** Attributes and levels included in the discrete choice experiment, including the names of the related dummy variables described in Equation 1.

Attribute	Levels	Dummy coding variable name (Equation 1)
Opening times	Only in the morning	[Reference category]
	Only in the afternoon	Afternoon
	Morning and afternoon	MorningAfternoon
	Morning, afternoon and evening	MorningAfternoonEvening
Weekend opening	Open every day	Weekend
	Closed at weekends	[Reference category]
How you can contact the medicines helpline	Phone	[Reference category]
	Phone or email	Email
	Phone or video call	Video
	Phone or text message	Text
How quickly you can talk to a pharmacist	Straight away	PharmacistImmediately
	Leave a message	[Reference category]
How quickly your question is answered	Less than an hour	AnswerHour
	Same day	AnswerSameDay
	Next day	[Reference category]
Where the medicines helpline is based	At your hospital	Local
	**Not** at your hospital	[Reference category]
Cost to the NHS	£10 per call	Continuous variable named ‘Cost’
	£20 per call	

### Experimental design

In line with good practice in health research, we constructed the attributes and levels into full profile tasks without an opt-out or status-quo option (Bridges et al., 2011). It is good practice to limit participant burden by including only 8–16 choice sets in a DCE (Bridges et al., 2011), so we used Stata (v16) to produce a D-efficient design with 20 choice sets divided between two blocks of ten. We confirmed this achieved level balance for all attributes and avoided dominant pairs, implausible combinations and co-linearity (assessed using Kendall’s Tau-b) (Bridges et al., 2011).

### Instrument design

We incorporated the final experimental design into an online survey using Jisc Online Surveys (www.onlinesurveys.ac.uk) (Supplemental Material, Appendix 3). Two versions of the survey were prepared, identical except that they contained either the block 1 or 2 choice tasks. They began with screening questions to confirm participant eligibility. The next section introduced a scenario that participants were asked to consider while completing the DCE (Box 1), followed by an explanation of each of the attributes and its levels. To familiarise participants with the attributes, each explanation was followed by a question about its importance; there was no intention to analyse these responses (Bridges et al., 2011). The concept of a DCE choice task was then explained, including an example question and a restatement of the scenario (Box 1) (Bridges et al., 2011), before presenting the ten choice tasks. The order of these choices sets and the attributes within each set were randomised (Bridges et al., 2011). To test the validity of participants’ DCE responses, the next section included questions about understanding of the choice tasks and the frequency with which each of the attributes was considered (Janssen et al., 2018; Porteous et al., 2016). The final section collected information on participants’ personal characteristics, using questions based on the 2011 UK Census (Office for National Statistics, 2011). The draft survey was pre-tested in cognitive interviews with four members of the public. This confirmed that participants could reasonably evaluate the full choice sets and that participant burden was acceptable, and led to minor clarifications throughout the survey.

Box 1: Hypothetical scenario that participants were asked to consider while completing the DCE.

**Table:** 

‘Imagine you (or someone you care for) have been treated in hospital for a few days and then go home. Whilst you were in hospital, something about your medicines was changed. The hospital provides a medicines helpline, which lets you contact a pharmacist if you have any questions about your medicines.’

### Participants and data collection

Participants aged ≥18 years, living in the UK and who regularly took one or more prescribed medicines (or who provided unpaid care to someone who did), were eligible. Participants were recruited via Research for the Future (www.researchforthefuture.org/), a National Institute of Health and Care Research-funded organisation that maintains a database of adults in England with an interest in health research participation. An invitation was sent to the 5,002 people in this database in January 2021; half linked to the block 1 survey and half to the block 2 survey. The invitation was also shared via the website and social media channels of Research for the Future. After completing the survey, participants were offered the opportunity to enter a draw to win one of three £20 shopping vouchers. The target sample size was at least 300 participants, as recommended by Orme (2019) and good practice guidelines (Bridges et al., 2011).

### Statistical analysis

Participant characteristics and validity question responses were summarised using descriptive statistics. The main effects model was estimated in Stata (v16) using conditional logistic regression to allow for multiple observations from individuals, with all variables except cost coded as dummy variables, with the least advantageous level as the ref category (Table 1). The systematic utility *V* of PMHS *j* was modelled as a linear and additive function of its attributes:

(1) $$\begin{array}{rl} & V_{j}=\alpha +{\text{β}}_{1}\mathrm{Afternoon}+{\text{β}}_{2}\mathrm{MorningAfternoon}\\ & +{\text{β}}_{3}\mathrm{MorningAfternoonEvening}+{\text{β}}_{4}\mathrm{Weekend}+{\text{β}}_{5}\mathrm{Email}+{\text{β}}_{6}\mathrm{Video}\\ & +{\text{β}}_{7}\mathrm{Text}+{\text{β}}_{8}\mathrm{PharmacistImmediately}+{\text{β}}_{9}\mathrm{AnswerSameDay}\\ & +{\text{β}}_{10}\mathrm{AnswerHour}+{\text{β}}_{11}\mathrm{Local}+{\text{β}}_{12}\mathrm{Cost} \end{array}$$

Here, α is a constant that captures mean effects of unobserved factors (Amaya-Amaya et al., 2008). Coefficients β_1_ to β_11_ are associated with categorical attributes coded as dummy variables (Table 1), so indicate the effect of the presence of that attribute on the overall PMHS utility (*V*). For example, β_1_ represents the effect on utility of the PMHS opening in the afternoon as opposed to the morning. In contrast, β_12_ represents the effect of a £1 increase in the cost of answering an enquiry. Utility and marginal willingness-to-pay for each attribute and level were calculated. Utility was estimated using Equation 1. Willingness-to-pay was estimated using Equation 2, where β_x_ is the attribute of interest:

(2) $$\mathrm{WTP}=\;\frac{{\text{β}}_{x}}{-{\text{β}}_{12}}$$

To investigate associations between participant characteristics and preferences, we used latent class models estimated using the LCLOGIT package in Stata (v16). Results are reported for three latent classes, as this minimised the consistent Akaike information criterion and the Bayesian information criterion when compared with models ranging from two to four classes. Models with five or more classes did not converge. Structural variables based on participants’ characteristics associated with the likelihood of subjects belonging to a specific class were identified. Except for age and number of medicines, these structural variables were coded as dummy variables and related to groups of at least 100 participants sharing a characteristic.

### Research ethics

This study was approved by the Research Ethics Approval Committee for Health at the University of Bath (ref: EP 19/20 062) in January 2021. Participant information was provided at the start of the survey (Supplemental Material, Appendix 3). Participants could only access the survey after agreeing with a statement that they had read this information and consented to participate.

## Results

### Participant characteristics

The survey was completed by 460 participants (block 1: 303; bock 2: 157), a response rate of 9.2% of email invitations. Participant characteristics are summarised in Table 2. There were similar numbers of male and female participants, from a wide range of ages. However, there were few participants from non-white ethnicities and three quarters lived in north-west England (reflecting the underlying membership of the Research for the Future database). Over half of participants had attended university and a similar proportion were retired.

**Table 2.** Participant characteristics (*n* = 460).

Gender, *n* (%)^1^	Female	245 (53.4%)
	Male	214 (46.6%)
	Prefer not to say	1
Age, years^2^	Mean (SD)	60.9 (12.6)
	Range	20–91
Ethnicity, *n* (%)^1^	White	437 (95.4%)
	Mixed/multiple ethnic groups	5 (1.1%)
	Asian/Asian British	8 (1.7%)
	Black/African/Caribbean/Black British	3 (0.7%)
	Other	5 (1.1%)
	Prefer not to say	2
UK region, *n* (%)^1^	London	10 (2.2%)
	South East England	24 (5.2%)
	South West England	30 (6.5%)
	East of England	13 (2.8%)
	East Midlands	7 (1.5%)
	West Midlands	15 (3.3%)
	Yorkshire and Humberside	14 (3.1%)
	North East England	5 (1.1%)
	North West England	341 (74.3%)
	Northern Ireland	0 (0%)
	Scotland	0 (0%)
	Wales	0 (0%)
	Prefer not to say	1
Highest level of education, *n* (%)^1^	GCSE/O-level or equivalent	90 (20.1%)
	A-level or equivalent	32 (7.1%)
	Vocational (e.g. NVQ)	54 (12.1%)
	University or other higher education	250 (55.8%)
	Other	22 (4.9%)
	Prefer not to say	12
Occupation, *n* (%)^1,3^	Working full-time	108 (23.5%)
	Working part-time	60 (13.1%)
	Unemployed	7 (1.5%)
	Looking after home or family	17 (3.7%)
	Full-time student	0 (0.0%)
	Part-time student	1 (0.2%)
	Retired	239 (52.1%)
	Long-term sick or disabled	44 (9.6%)
	Other	15 (3.3%)
	Prefer not to say	1
Number of regular prescribed medicines^4^	Mean (SD)	6.0 (4.8)
Last overnight hospital admission, *n* (%)^1^	Never	24 (5.2%)
	More than 5 years ago	230 (50.1%)
	1–5 years ago	160 (34.9%)
	Less than 1 year ago	45 (9.8%)
	Prefer not to say	1
Total annual household income, *n* (%)^1^	Up to £5,199	6 (1.8%)
	£5,200 and up to £10,399	25 (7.5%)
	£10,400 and up to £15,599	40 (12%)
	£15,600 and up to £20,799	41 (12.3)
	£20,800 and up to £25,999	34 (7.4%)
	£26,000 and up to £31,199	38 (10.2%)
	£31,200 and up to £36,399	35 (10.5%)
	£36,400 and up to £51,999	52 (15.6%)
	£52,000 and above	63 (18.9%)
	Prefer not to say	126

*n* = number of participants; SD = standard deviation.

^1^ The denominator for percentage calculations is the number of participants who chose to answer the question.

^2^ One participant chose not to disclose their age.

^3^ Participants were asked to select all occupations that applied, so total is greater than 460.

^4^ Nine participants chose not to disclose their number of medicines.

### Main effects model

Table 3 shows estimates of the main effects model. There were levels for all attributes that were significantly different to zero, suggesting that they all contributed to respondents’ preferences. Theoretical validity is demonstrated by the significant negative coefficient for cost, increasingly positive coefficients as opening times and days are increased, and positive coefficients for levels reflecting an enhanced service (e.g. additional contact methods or a faster response).

**Table 3.** Parameter coefficients and marginal willingness to pay estimated for the main effects model.

Variable	Regression coefficient		Marginal willingness to pay	
	Coefficient (95% CI)	*P*-value	Willingness to pay (95% CI)	*P*-value
Opening times (reference category: morning only)	[0]			
Afternoon only	0.078 (−0.076, 0.232)	0.320	£0.91 (−0.86, 2.68)	0.314
Morning and afternoon	0.673 (0.522, 0.825)	<0.001	£7.83 (6.01, 9.66)	<0.001
Morning, afternoon and evening	0.764 (0.626, 0.902)	<0.001	£8.89 (7.22, 10.56)	<0.001
Weekend opening (reference category: not open weekends)	[0]			
Open at weekends	0.962 (0.878, 1.046)	<0.001	£11.20 (10.05, 12.34)	<0.001
Contact methods (reference category: telephone only)	[0]			
Telephone or email	0.467 (0.328, 0.607)	<0.001	£5.44 (3.67, 7.21)	<0.001
Telephone or online video call	0.235 (0.107, 0.362)	<0.001	£2.73 (1.23, 4.23)	<0.001
Telephone or text messages	0.383 (0.235, 0.532)	<0.001	£4.46 (2.74, 6.18)	<0.001
Speed of pharmacist contact (reference category: leave a message)	[0]			
Talk to a pharmacist immediately	0.487 (0.404, 0.570)	<0.001	£5.67 (4.51, 6.83)	<0.001
Speed of answer (reference category: next day)	[0]			
Same day	0.797 (0.678, 0.916)	<0.001	£9.27 (7.67, 10.88)	<0.001
Less than an hour	0.717 (0.600, 0.834)	<0.001	£8.34 (6.77, 9.91)	<0.001
Location of helpline (reference category: not at your hospital)	[0]			
At your hospital	0.299 (0.219, 0.379)	<0.001	£3.48 (2.46, 4.50)	<0.001
Cost to the NHS, £	−0.086 (−0.094, −0.077)	<0.001	-	-
Goodness of fit				
Number of observations	9200			
Numbers of choices	4600			
Log likelihood	−2248			

95% CI = 95% confidence interval.

Marginal willingness-to-pay (Table 3) demonstrated that the most important attributes for participants were receiving an answer on at least the same day and a PMHS open at weekends and in the morning, afternoon and evening. Use of video calling and being located at the participant’s hospital significantly increased preferences, but were the least valued attributes.

Most participants (93%) reported understanding the concept of making choices and only 18% found this confusing (Table 4). A minority (17%) reported needing more information and 74% found the PMHS options made sense. The majority (≥85%) of respondents reported sometimes or always considering all the attributes apart from PMHS location (53%) (Table 5).

**Table 4.** Participants’ responses to statements related to the ease with which they could complete the survey (*n* = 460).

*n* (%)	Strongly disagree	Disagree	Neither agree nor disagree	Agree	Strongly agree
I understood the concept of making choices between different helplines	7 (1.5%)	6 (1.3%)	21 (4.6%)	186 (40.4%)	240 (52.5%)
I found making a choice between different helplines confusing	94 (20.4%)	185 (40.2%)	100 (21.7%)	66 (14.3%)	15 (3.3%)
When choosing between different helplines I needed more information than was provided	72 (15.7%)	193 (42.0%)	119 (25.9%)	53 (11.5%)	23 (5.0%)
I found that the available helpline options made sense	14 (3.0%)	33 (7.2%)	75 (16.3%)	208 (45.2%)	130 (28.3%)

**Table 5.** Participants’ self-assessment of how often they considered each of the attributes when answering choice questions.

*n* (%)	Never considered	Sometimes considered	Always considered
Opening times	17 (3.7%)	132 (28.7%)	311 (67.6%)
Weekend opening	27 (5.9%)	172 (37.4%)	261 (56.7%)
How you can contact the helpline	57 (12.4%)	180 (39.1%)	223 (48.5%)
How quickly you can talk to a pharmacist	24 (5.2%)	199 (43.3%)	237 (51.5%)
How quickly your question is answered	20 (4.3%)	155 (33.7%)	285 (62.0%)
Where the medicines helpline is based	215 (46.7%)	149 (32.4%)	96 (20.9%)
Cost to the NHS	70 (15.2%)	183 (39.8%)	207 (45.0%)

### Latent class model

Table 6 shows the preferences estimated for the three latent classes. Two structural variables were identified as significantly associated with membership of these classes (Table 7): gender and working full-time. Class 1 valued longer opening hours, weekend opening and a quick answer more strongly than other attributes and more strongly than the main effects model. Membership of this class was significantly associated with female gender and working full-time. Only three attributes significantly influenced the preferences of class 2. Being located at the participant’s hospital was the most strongly valued, over three times more than the main effects model. Weekend opening was also valued, but counterintuitively, longer opening hours decreased participants’ preferences. Membership of this class was significantly associated with female gender and working full-time. The preferences of class 3 were similar to the main effects model, with more equal weight given to a variety of attributes. This group most strongly valued a fast reply.

**Table 6.** Estimated preference parameters of the latent-class model.

Variable	Class 1		Class 2		Class 3	
	Coefficient (95% CI)	*P*-value	Coefficient (95% CI)	*P*-value	Coefficient (95% CI)	*P*-value
Opening times (reference category: morning only)	[0]		[0]		[0]	
Afternoon only	1.266 (0.510, 2.021)	0.001	−0.869 (−1.639, −0.098)	0.027	−0.167 (−0.576, 0.242)	0.424
Morning and afternoon	3.038 (1.714, 4.361)	<0.001	−0.720 (−1.378, −0.062)	0.032	0.668 (0.324, 1.012)	<0.001
Morning, afternoon and evening	4.223 (2.456, 5.990)	<0.001	−0.743 (−1.345, −0.141)	0.016	0.693 (0.354, 1.032)	<0.001
Weekend opening (reference category: not open weekends)	[0]		[0]		[0]	
Open at weekends	2.572 (2.118, 3.026)	<0.001	0.402 (0.034, 0.770)	0.032	0.667 (0.442, 0.891)	<0.001
Contact methods (reference category: telephone only)	[0]		[0]		[0]	
Telephone or email	−0.479 (−1.046, 0.088)	0.098	0.751 (−0.058, 1.560)	0.069	0.915 (0.580, 1.250)	<0.001
Telephone or online video call	0.920 (−0.177, 2.017)	0.100	−0.145 (−0.621, 0.331)	0.551	0.104 (−0.167, 0.374)	0.451
Telephone or text messages	1.202 (0.584, 1.820)	<0.001	1.727 (−0.310, 3.764)	0.097	−0.216 (−0.635, 0.204)	0.313
Speed of pharmacist contact (reference category: leave a message)	[0]		[0]		[0]	
Talk to a pharmacist immediately	1.146 (0.611, 1.681)	<0.001	0.271 (−0.027, 0.568)	0.075	0.856 (0.602, 1.110)	<0.001
Speed of answer (reference category: next day)	[0]		[0]		[0]	
Same day	3.909 (2.314, 5.504)	<0.001	0.084 (−0.465, 0.632)	0.765	1.089 (0.772, 1.406)	<0.001
Less than an hour	2.441 (1.521, 3.360)	<0.001	0.509 (−0.119, 1.136)	0.112	1.121 (0.809, 1.434)	<0.001
Location of helpline (reference category: not at your hospital)	[0]		[0]		[0]	
At your hospital	−0.387 (−0.848, 0.074)	0.100	0.976 (0.069, 1.883)	0.035	0.464 (0.258, 0.670)	<0.001
Cost to the NHS, £	−0.210 (−0.297, −0.123)	<0.001	−0.284 (−0.389, −0.178)	<0.001	0.001 (−0.028, 0.029)	0.971

95% CI = 95% confidence interval.

**Table 7.** Estimated class membership parameters (in reference to class 3) of the latent-class model. The class share of class 3 was 0.309.

Variable	Class 1		Class 2	
	Class share: 0.427		Class share: 0.265	
	Coefficient (95% CI)	*P*-value	Coefficient (95% CI)	*P*-value
Female gender	1.159 (0.444, 1.874)	0.001	0.730 (0.035, 1.425)	0.040
Age	0.035 (−0.007, 0.077)	0.101	0.038 (−0.001, 0.077)	0.059
Home in the north-west of England	−0.511 (−1.401, 0.379)	0.260	−0.383 (−1.234, 0.468)	0.378
Highest level of education: university	−0.656 (−1.443, 0.131)	0.102	−0.634 (−1.406, 0.138)	0.108
Highest level of education: GCSE	−0.147 (−1.218, 0.923)	0.787	−0.165 (−1.186, 0.855)	0.751
Occupation: working full-time^a^	1.428 (0.303, 2.554)	0.013	1.312 (0.255, 2.369)	0.015
Occupation: retired^a^	0.351 (−0.727, 1.430)	0.523	0.342 (−0.641, 1.324)	0.495
Number of regular prescribed medicines	−0.013 (−0.076, 0.05)	0.691	−0.029 (−0.099, 0.042)	0.425
Overnight hospital admission in the past 5 years	0.177 (−0.536, 0.890)	0.626	−0.247 (−0.935, 0.441)	0.482
Total annual household income: less than £20,799	0.552 (−0.363, 1.467)	0.237	0.454 (−0.451, 1.36)	0.325
Total annual household income: £20,800 to £36,399	0.199 (−0.802, 1.201)	0.697	0.081 (−0.887, 1.049)	0.870
Total annual household income: more than £36,400	0.075 (−1.027, 1.178)	0.893	0.063 (−0.891, 1.018)	0.897

^a^ Participants were asked to select all occupations that applied.

95% CI = 95% confidence interval.

## Discussion

All the included attributes influenced participants’ preferences for PMHS provision, with weekend opening, longer opening hours and a fast answer being most highly valued. However, one latent class containing approximately a quarter of participants most strongly valued the PMHS being located at their own hospital. It is unsurprising that full-time work was associated with much stronger preferences for longer opening hours and weekend opening in latent class 1, as these would enable easier access to support outside of working hours. There was no evidence that UK region, educational level, number of medicines and time since last hospital admission affected preferences, although there were limited numbers of participants in some of these categories.

Only one previous study has examined the importance of different PMHS attributes. In this survey of 75 recent PMHS enquirers (O'Grady et al., 2021), 75% of participants stated that being able to contact the PMHS easily was extremely important, aligned with the importance of opening days and hours in the present study. However, in contrast only 57–58% of participants in the former study stated that agreeing and meeting a deadline for an answer to their question was extremely important (O'Grady et al., 2021), in contrast to the present study where a speedy answer was as important as ease of contact. Similarly, a qualitative interview study with 40 PMHS enquirers (Williams et al., 2020b) reported that they described needing timely and easy access to advice, and quick resolution of problems. Interviewees also suggested PMHS could be improved by extending their opening hours (Williams et al., 2020b), which aligns with the importance of opening times and speed of answer in the present study.

A recent systematic review reported that a mean of 85% of PMHS enquirers found it easy to access the service and 94% received information in a timely manner (Williams et al., 2019b), suggesting that existing PMHS are meeting enquirers’ needs. However, there may be patients who are unable to contact a PMHS due to limited opening hours, as only 7% of PMHS in England open in the evenings and weekends (Williams et al., 2018), attributes that were highly valued by participants in the present study. Less than half of English PMHS offered alternative means of contact to the telephone in 2017 (Williams et al., 2018), which aligns with the relatively lower value given to such attributes in the present study.

In 2017, only 3% NHS Trusts in England reported providing a PMHS via another organisation, but a study of PMHS providers’ views of the future suggested that due to limited resources, improvements such as extended opening hours might only be possible via a regional or national service (Williams et al., 2021). This is aligned with the relatively low value placed on a local service in the main effects model, but it should be noted that a local PMHS was the most valued attribute for one of the latent classes containing over a quarter of participants.

Strengths of this study include the first use of a DCE to investigate potential enquirers’ preferences for any type of medicines information service. This allowed analysis of the strength of preferences and how they were traded off, which is not possible from the small amount of previous research in this area. In addition, this study achieved a large sample size and recruited potential PMHS enquirers rather than past enquirers, so included the views of people who might otherwise have been excluded by the characteristics of existing PMHS. Potential attributes were identified from an extensive programme of mixed methods research and documentary analysis, and prioritised with input from stakeholders. Most participants stated that they understood the survey, had sufficient information and considered most of the attributes in their decision making (Table 3 and Table 4). The only exception was the PMHS location attribute, which may reflect how relatively unimportant this was to many participants. The main limitations of this study relate to the characteristics of the sample, with non-white ethnicities being under-represented and university educated people in the north-west of England being over-represented. In addition, the survey was completed online, thus excluding people without internet access which is likely to affect how they access medicines information. These limitations limit the generalisability of the findings and the understanding of how preferences are associated with varying personal characteristics.

Patient convenience is often critical to healthcare utilisation, but these findings allow PMHS providers to identify those aspects of convenience that are most valued by potential enquirers. For example, they suggest that PMHS providers should prioritise opening in mornings and afternoons, and at weekends, and answering questions on the same day, because participants placed limited additional value on other aspects of convenience, such as evening opening or receiving an answer in less than an hour. Regional or national PMHS with access to the necessary patient information may be acceptable to enquirers, but achieving this requirement may be challenging (Williams et al., 2021). Future research should address whether such changes increase the reach of PMHS and improve outcomes, and investigate the preferences of a nationally representative sample. The present study has demonstrated the utility of a DCE in this field of research, so a similar study of professional enquirers to medicines information services would be useful to enable providers to tailor their services to users’ needs.

## Conclusion

Potential enquirers to PMHS most highly value weekend opening, longer opening hours and provision of a quick answer, and variation in preferences is associated with gender and full-time work. PMHS providers should prioritise these service characteristics to make most efficient use of their available resources in meeting patient need.

## Supplementary Material

Supplemental Material
