# Supplementary material for: Patient preferences for the provision of NHS medicines helpline services: a discrete choice experiment
Source: J Pharm Policy Pract. 2024 Oct 1;17(1):2404973. doi: 10.1080/20523211.2024.2404973 (PMC11445913; doi:10.1080/20523211.2024.2404973)
Supplement: Supplemental Material [file JPPP_A_2404973_SM0065.docx]

# Journal of Pharmaceutical Policy and Practice

# Patient preferences for the provision of NHS medicines helpline services: a discrete choice experiment

# Supplementary Material

Ben Ashby (ORCID 0009-0002-3628-4052)^1^ and Matthew D. Jones (ORCID 0000-0002-2617-4098)^2^*

^1^Institute for Mathematical Innovation, University of Bath, Bath, UK.

^2^Department of Life Sciences, University of Bath, Bath, UK.

*Corresponding author:

Department of Life Sciences, University of Bath, Bath, BA2 7AY, UK.

Telephone: +44 1225 383829, E-mail: [M.D.Jones@bath.ac.uk](mailto:M.D.Jones@bath.ac.uk)

**Appendix 1: the Conjoint Analysis Applications in Health Checklist**

Bridges JF, Hauber AB, Marshall D, et al. Conjoint analysis applications in health - a checklist: a report of the ISPOR Good Research Practices for Conjoint Analysis Task Force. Value in Health 2011; 14: 403-413. DOI: 10.1016/j.jval.2010.11.013.

|  | **Notes or section in manuscript where discussed** |
| --- | --- |
| 1. Was a well-defined research question stated and is conjoint analysis an appropriate method for answering it? | |
| 1.1 Were a well-defined research question and a testable hypothesis articulated? | Introduction, paragraph 4 |
| 1.2 Was the study perspective described, and was the study placed in a particular decision-making or policy context? | Introduction, paragraphs 1-2 |
| 1.3 What is the rationale for using conjoint analysis to answer the research question? | Introduction, paragraph 3 |
| 2. Was the choice of attributes and levels supported by evidence? | |
| 2.1 Was attribute identification supported by evidence (literature reviews, focus groups, or other scientific methods)? | Attributes and Levels |
| 2.2 Was attribute selection justified and consistent with theory? | Attributes and Levels, Appendix 2 |
| 2.3 Was level selection for each attribute justified by the evidence and consistent with the study perspective and hypothesis? | Attributes and Levels, Appendix 2 |
| 3. Was the construction of tasks appropriate? | |
| 3.1 Was the number of attributes in each conjoint task justified (that is, full or partial profile)? | Experimental Design, Instrument Design (pre-testing) |
| 3.2 Was the number of profiles in each conjoint task justified? | Experimental Design |
| 3.3 Was (should) an opt-out or a status-quo alternative (be) included? | Experimental Design |
| 4. Was the choice of experimental design justified and evaluated? | |
| 4.1 Was the choice of experimental design justified? Were alternative experimental designs considered? | Experimental Design |
| 4.2 Were the properties of the experimental design evaluated? | Experimental Design |
| 4.3 Was the number of conjoint tasks included in the data-collection instrument appropriate? | Experimental Design |
| 5. Were preferences elicited appropriately, given the research question? | |
| 5.1 Was there sufficient motivation and explanation of conjoint tasks? | Instrument Design |
| 5.2 Was an appropriate elicitation format (that is, rating, ranking, or choice) used? Did (should) the elicitation format allow for indifference? | Instrument Design |
| 5.3 In addition to preference elicitation, did the conjoint tasks include other qualifying questions (for example, strength of preference, confidence in response, and other methods)? | Instrument Design |
| 6. Was the data collection instrument designed appropriately? | |
| 6.1 Was appropriate respondent information collected (such as sociodemographic, attitudinal, health history or status, and treatment experience)? | Instrument Design |
| 6.2 Were the attributes and levels defined, and was any contextual information provided? | Instrument Design |
| 6.3 Was the level of burden of the data-collection instrument appropriate? Were respondents encouraged and motivated? | Experimental Design, Instrument Design (pre-testing), Participants and Data Collection (prize draw), Appendix 3 (regular encouragement throughout the survey) |
| 7. Was the data-collection plan appropriate? | |
| 7.1 Was the sampling strategy justified (for example, sample size, stratification, and recruitment)? | Participants and Data Collection |
| 7.2 Was the mode of administration justified and appropriate (for example, face-to-face, pen-and-paper, web-based)? | An online survey was used to reach a large sample with limited resources. The resultant limitations described in the Discussion. |
| 7.3 Were ethical considerations addressed (for example, recruitment, information and/or consent, compensation)? | Research Ethics |
| 8. Were statistical analyses and model estimations appropriate? | |
| 8.1 Were respondent characteristics examined and tested? | Participant Characteristics, Table 2 |
| 8.2 Was the quality of the responses examined (for example, rationality, validity, reliability)? | Main Effects Model, Tables 4 and 5 |
| 8.3 Was model estimation conducted appropriately? Were issues of clustering and subgroups handled appropriately? | Statistical Analysis |
| 9. Were the results and conclusions valid? | |
| 9.1 Did study results reflect testable hypotheses and account for statistical uncertainty? | Main Effects Model, Latent Class Model |
| 9.2 Were study conclusions supported by the evidence and compared with existing findings in the literature? | Discussion paragraphs 1-4 |
| 9.3 Were study limitations and generalizability adequately discussed? | Discussion paragraph 5 |
| 10. Was the study presentation clear, concise, and complete? | |
| 10.1 Was study importance and research context adequately motivated? | Introduction paragraphs 1-3 |
| 10.2 Were the study data-collection instrument and methods described? | Methods, Appendix 3 |
| 10.3 Were the study implications clearly stated and understandable to a wide audience? | Discussion paragraph 6 |

**Appendix 2: information on potential attributes and levels shared with pharmacists who currently operated a medicines helpline**

| **Research question (RQ):** | |
| --- | --- |
| What are preferences of people who regularly take at least one medicine for different attributes related to the delivery of an NHS medicines helpline which might affect their choice on whether to use the service? | |
|  |  |
| **Background** |  |
| This document describes the selection of attributes and levels for a discrete choice experiment to answer this research question | |
| The discrete choice experiment will be a survey that asks people to choose their preferred helpline configuration from each of a series of pairs of possible configurations | |
| The helpline configurations will be described using attributes and levels | |
| Attributes are a characteristic of how a helpline is operated that can be varied by the staff running the helplines, e.g. opening hours per day | |
| Levels describe the different values that each attribute might take, e.g. opening for 4, 8 or 12 hours/day | |
| Each pair of configurations will consist of all the attributes selected for the study, but with different combinations of levels, e.g: | |

|  | **Opening hours/day** |
| --- | --- |
| **Helpline A** | 8 |
| **Helpline B** | 4 |

| Participants are then asked to choose if they prefer helpline A or B - so in this case they must decide if they prefer longer daily opening hours or opening at weekends more. | |
| --- | --- |
| Once enough people have stated their preferences for enough of these pairs, mathematical analysis can be used to work out how much people prefer the different attributes compared to each other | |
|  |  |
| Therefore, the selection of attributes is critical, as they need to be relevant to the research question, relate to things that people are likely to value and easy to understand | |
| Levels also need to be selected carefully to be realistic and not extreme | |
|  |  |
| **Notes on attribute selection:** | |
| Selected attributes need to be relevant to the research question (attributes which might affect the decision to use the service) but also to staff deciding how to operate a helpline. | |
| The research question relates to attributes which might affect a patient's choice on whether to use the service | |
| Therefore, for new callers these attributes need to be obvious from advertising etc | |
| However, repeat callers might also be influenced by their previous calls, so aspects of the experience of calling are also potentially important | |
|  |  |
| **What I'd like you to do** |  |
| Below are a list of all the possible attributes I identified from the latest relevant literature | |
| The first part lists the attributes I have initially decided to include, with their related levels and justifications for these decisions | |
| The second part lists the attributes I have initially decided to exclude, with a justification | |
| Could you look at these lists and think about whether these decisions and descriptions are appropriate from your perspective of someone running a helpline. There may also be other attributes I have not considered. | |

| **Included attributes** | **Justification for selection** | **Levels** | **Justification for levels** | **Links to other attributes** | **Source of attribute/levels** |
| --- | --- | --- | --- | --- | --- |
| Opening hours per day | New callers aware and feasible it will affect use. Relevant to people running a helpline. Variable between current helplines | 4, 8 or 12 hours/day | National standards have 4 or 8 hours. 86% already do 4hrs, 57% do 8hrs, 3% do evenings (i.e. 12 hours) | Days opening | 1,2,3,5 |
| Days opening per week | New callers aware and feasible it will affect use. Relevant to people running a helpline. Variable between current helplines | 5 or 7 days/week | National standards have 5 days, with weekends as commended. 96% already do 5 days, 5% do 7 days. Seems unlikely someone would do 6 days | Hours opening | 1,2,3,5 |
| Other means of access, e.g. email, webform, in person | New callers aware and feasible it will affect use. Relevant to people running a helpline. Only 39% do this | Phone only, phone+email, phone+video, phone+messaging service (SMS, WhatsApp) | Phone is the current baseline for comparison, email is widely used & mentioned by patients, video is potentially more relevant post-Covid, messaging services widely used. Ignored dedicated app (less likely in the near future), social media (IG concerns), webform (harder to explain on a survey), in person (unlikely to be used often) | Pharmacist availability | 1,2,3,5 |
| Contact with a pharmacy professional always available in advertised hours | New caller would not necessarily be aware before calling, but might hang up on voicemail. Relevant to people running a helpline. In standards and important to pts in (3) | "Calls answered immediately by pharmacy staff" vs "Callers leave an answerphone message and pharmacy staff phone them back later" | Calls answered immediately as the baseline comparator, answerphone as the common way to deal with non-available staff | Method of contact | 1,3,5 |
| Location of helpline (e.g. local or regional) | Potential policy question going forward (see (6)). New called might be aware and contacting own hospital important to patients in (3), so might affect decision to call | "Helpline is based at the hospital which treated you" vs "Helpline is **not** based at the hospital which treated you". | From (3), key characteristics seems to be going back to the place which treated you, hence 'hospital which treated you' and not at this hospital. Risk of people not reading 'not', but alternative wordings (e.g. helpline is located in another part of the country) do not necessarily relate to the hospital which treated you if people travel to hospital (rural areas, tertiary referral). Need to decide whether to specify that away from own hospital may now have hospital records access - contacting local hospital would delay, so links to speed of answer attribute. | Speed of reply | 2,4,6 |
| Feedback time | New callers would not be aware, but previous callers would & might influence decision to call again, as strongest preference in (2) and important in (3). Also relevant to people running helplines | Within an hour, same day, next day | Unlikely to be seen as good practice to let patient question remain unresolved for longer. Do not want to describe all levels with a number of hours, due to calls close to closing time | Location | 2,3,4 |
| Cost of call to the NHS | Needed to enable calculation of willingness for NHS to pay (which enables easy comparison on attributes and to other studies) | £10, £20, £30 | Technician answering a 15 min enquiry = £8.50, 8a pharmacist answering a 30 min enquiry £32. Rounded to ease cognitive burden on participants & added mid point. PSSRU 2019 data (which include salary, estates costs, capital overheads etc) give £34/hour for band 5 professional working in shared facilities & £64/hour for band 8a professional in shared facilities. |  | 2,7 |

| **Excluded attributes** | **Justification for exclusion** | **Source of attribute** |
| --- | --- | --- |
| Direct dial phone line | 97% already do this (5), so not relevant to people running helplines | 1 |
| Dedicated phone number | Callers would not be aware who else uses the number | 1 |
| Local rate or freephone number (not premium) | 99% already do this (5), so not relevant to people running helplines | 1 |
| Answerphone for OOH or if engaged | New caller would not necessarily be aware. 81% already do this (5), so less relevant to people running helplines. Good practice to do with minimal cost, so not a priority area | 1 |
| Promotion by methods agreed with local patients | New caller would not be aware that consultation had happened. Better advertising might increase helpline use, but the attribute is about consultation on the advertising | 1 |
| Promoted at all sites | New caller would not be aware | 1 |
| Promotion includes access times & example questions | Not an important question for helpline staff - easy to implement with little resource | 1 |
| Promoted to outpatients | Not all new callers would be aware. 84% already doing this (5), so less relevant to helpline staff | 1 |
| Additional promotion methods | Not all new callers would be aware. Vague attribute | 1 |
| All enquiries documented | Good practice, so cannot be influenced by research. New callers not aware | 1 |
| SOPs to advise staff | Good practice, so cannot be influenced by research. New callers not aware | 1 |
| Enquiries answers by competent staff | Good practice, so cannot be influenced by research. New callers not aware | 1 |
| Access to specified resources | Good practice, so cannot be influenced by research. New callers not aware | 1 |
| Access to patient's records at the hospital | Potentially an important question, and important to patients (3). But callers would not be aware. Could subsume into helpline location question | 1 |
| Hosted by an MI centre | Participants unlikely to know what an MI centre is, or how it compares to alternative locations, so not relevant to research question | 1 |
| Common Q&As on website | Might affect decision to call, but only if patient's question was answered online | 1 |
| Access to local combined health record | New caller would not be aware | 1 |
| Standards agreed with patients | New caller would not be aware | 1 |
| Assessment of satisfaction ongoing | New caller would not be aware | 1 |
| Feedback errors to trust | New caller would not be aware | 1,4 |
| Service improvement work | New caller would not be aware | 1 |
| User survey designed with patients | New caller would not be aware | 1 |
| Profession of staff working the helpline (e.g. nurse, pharmacist) | Not likely to be good practice to use a nurse, so not relevant to helpline staff. New caller unlikely to be aware of difference between pharm and tech. No significant effect in (2) | 2 |
| Helpline staff have "positive personal qualities" | Not known to a new caller, cannot be easily defined in a survey | 3 |
| Helpline is available to general public as well as patients | Research question is about patients - they are the main people we want to benefit | 5 |
| Advertising of helpline improved | Raises awareness of the service, does not change how it is valued by potential users | 3 |
| Helpline actively contacts patients after discharge | New service, so outside scope of RQ | 3,4 |
| Community pharmacy as initial point of contact for the helpline | New service, so outside scope of RQ | 3,4 |

| **References** |
| --- |
| 1) Wills S. Medicines helpline for hospital patients: national standard, www.ukmi.nhs.uk/filestore/ukmiacg/MedicinesHelplineStandardsvn3_2.pdf (2014, accessed 1st December, 2015 2015). |
| 2) Unpublished student pilot study. |
| 3) Williams M, Jordan A, Scott J, et al. Service users' experiences of contacting NHS patient medicines helpline services: a qualitative study. BMJ Open 2020; 10: e036326. 20200628. DOI: 10.1136/bmjopen-2019-036326. |
| 4) Williams M, Jordan A, Scott J, et al. Pharmacy professionals' experiences and perceptions of providing NHS patient medicines helpline services: a qualitative study. BMC Health Serv Res 2020; 20: 364. DOI: 10.1186/s12913-020-05182-w. |
| 5) Williams M, Jordan A, Scott J, et al. Operating a patient medicines helpline: a survey study exploring current practice in England using the RE-AIM evaluation framework. BMC Health Serv Res 2018; 18: 868. DOI: 10.1186/s12913-018-3690-9. |
| 6) Williams M, Jordan A, Scott J, et al. Pharmacy professionals' views regarding the future of NHS patient medicines helpline services: a multimethod qualitative study. BMC Health Serv Res 2021; 21: 137. 2021/02/14. DOI: 10.1186/s12913-021-06144-6. |
| 7) Curtis L and Burns A. Unit Costs of Health and Social Care 2018. 2018. Personal Social Services Research Unit, University of Kent, Canterbury. |
|  |
| Two systematic reviews also consulted, but no attributes identified: |
|  |
| Williams M, Jordan A, Scott J, et al. Examining the characteristics of users of NHS patient medicines helpline services, and the types of enquiries they make: a systematic review protocol. Eur J Hosp Pharm 2019. DOI: 10.1136/ejhpharm-2019-002001. |
| Williams M, Jordan A, Scott J, et al. A systematic review examining the effectiveness of medicines information services for patients and the general public. Int J Pharm Prac 2019 2019/09/13. DOI: 10.1111/ijpp.12571. |

**Appendix 3: the online survey for block 1 of the experimental design. The block 2 survey was identical except that it contained the block 2 choice sets.**

**
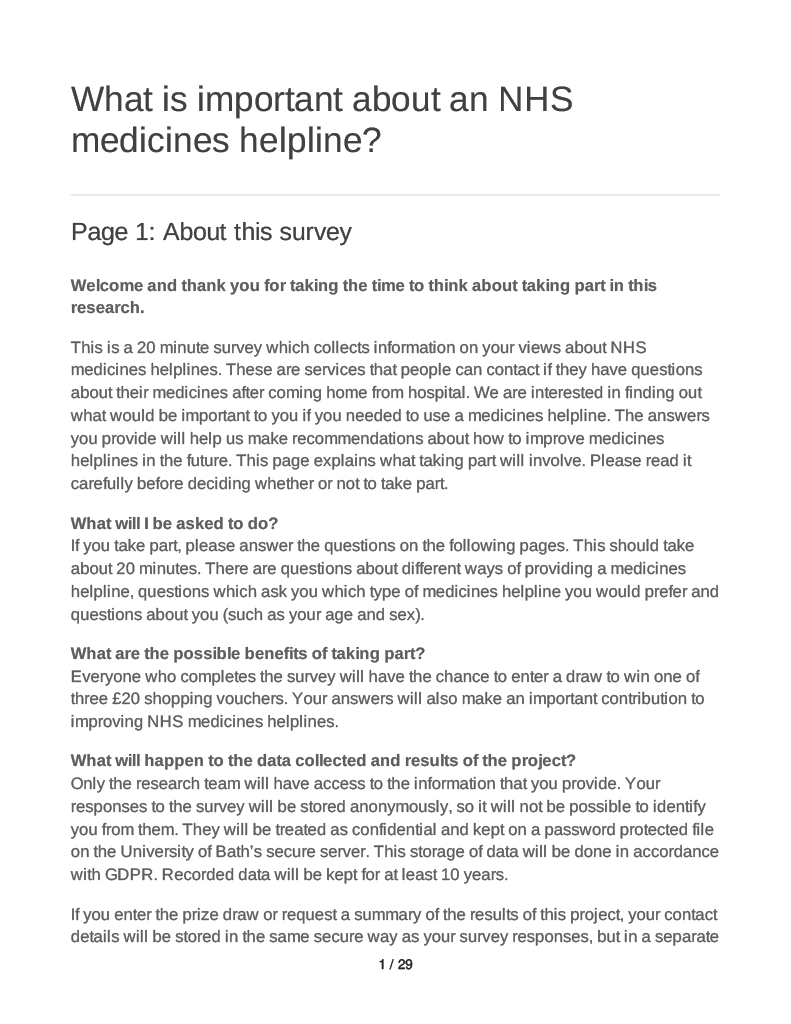
**

**
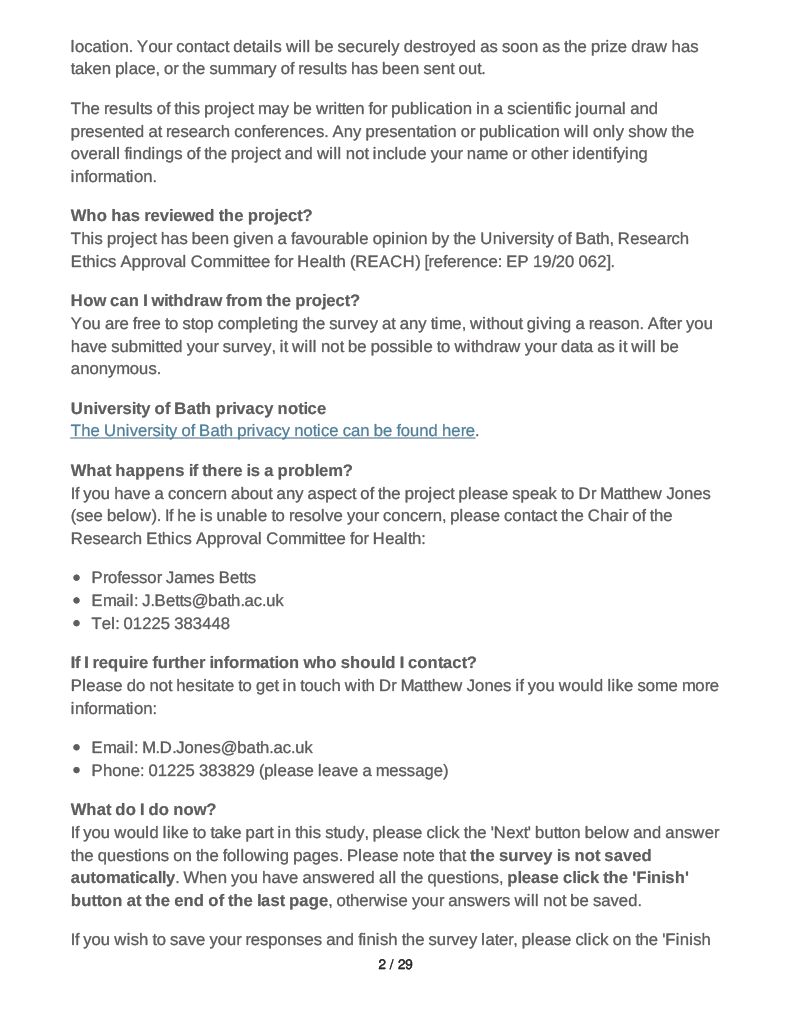
**

**
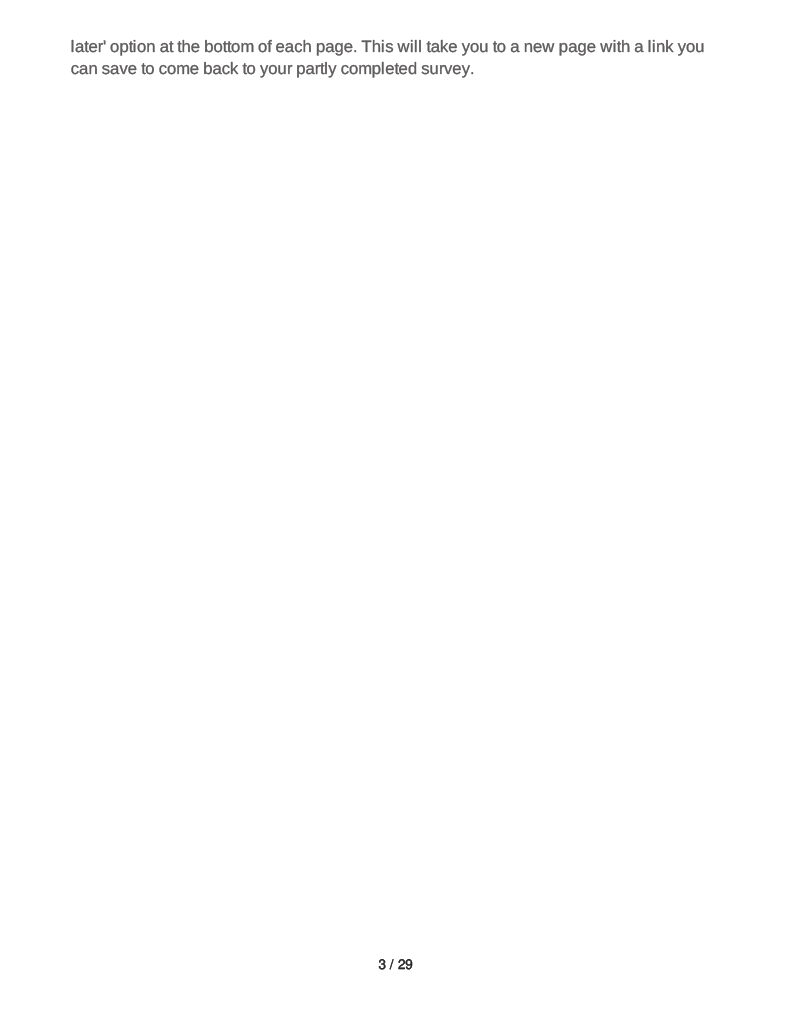
**

**
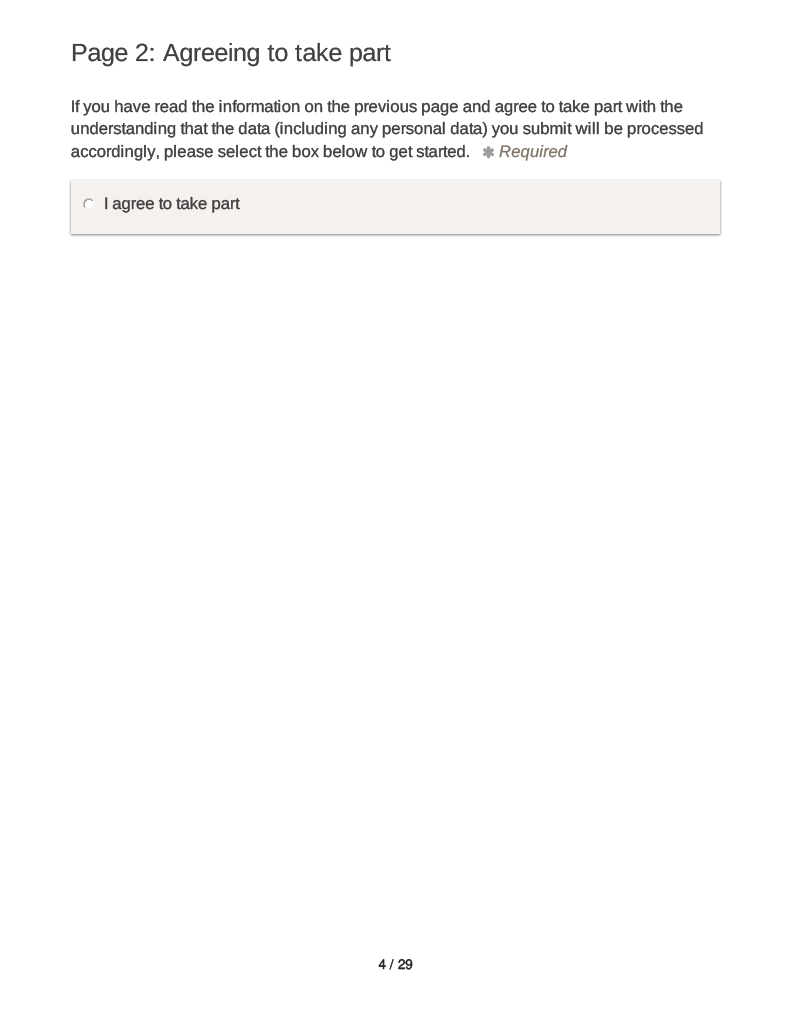
**

**
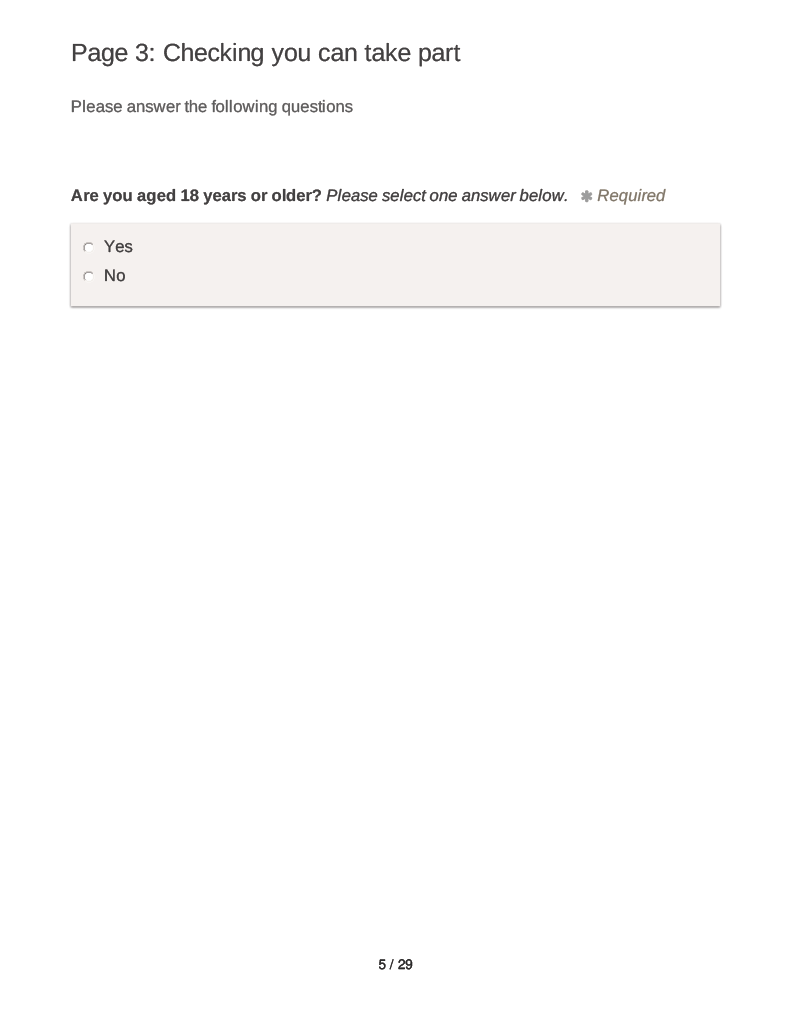
**

**
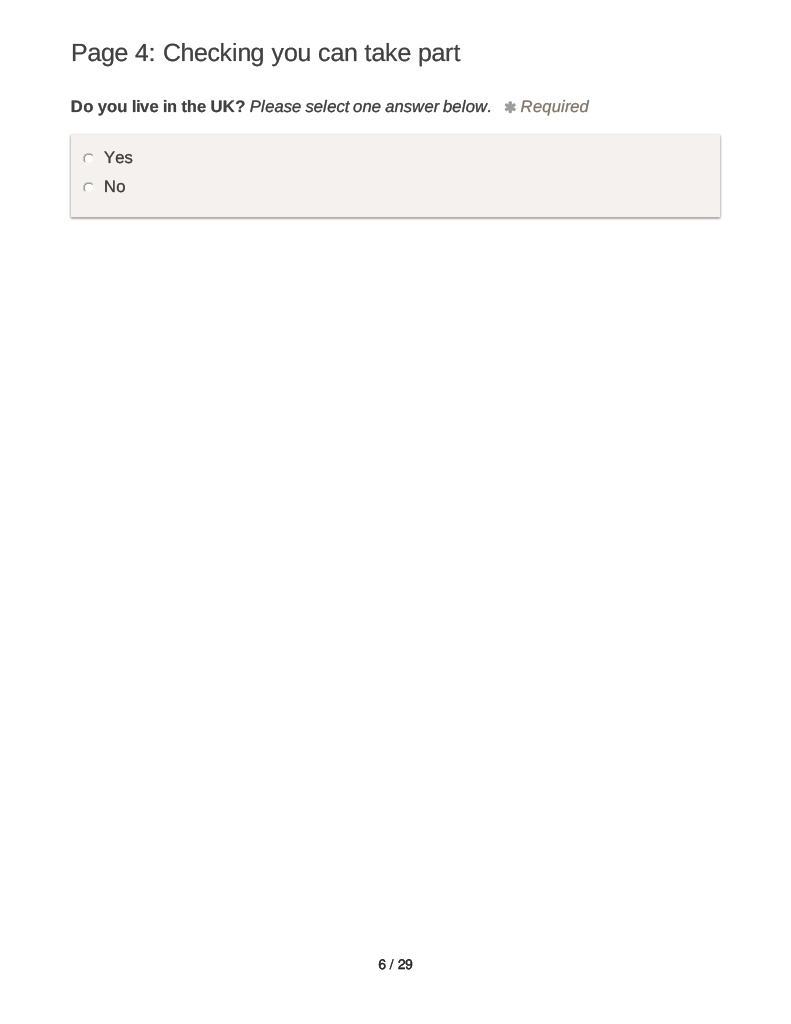
**

**
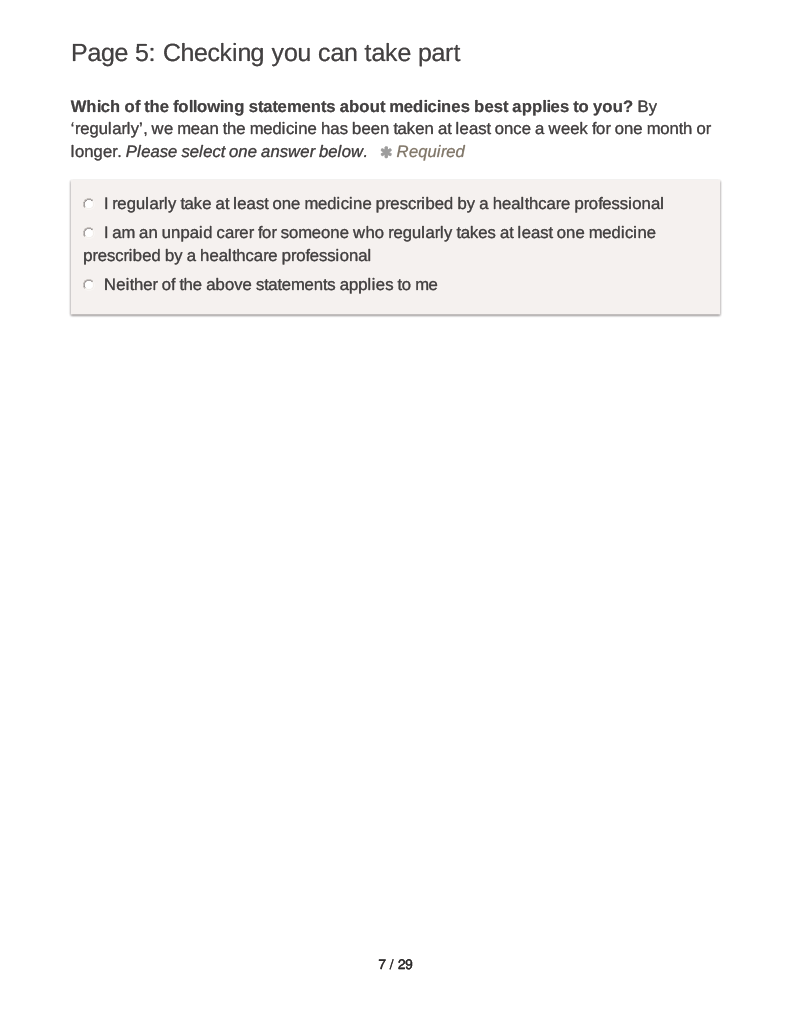
**

**
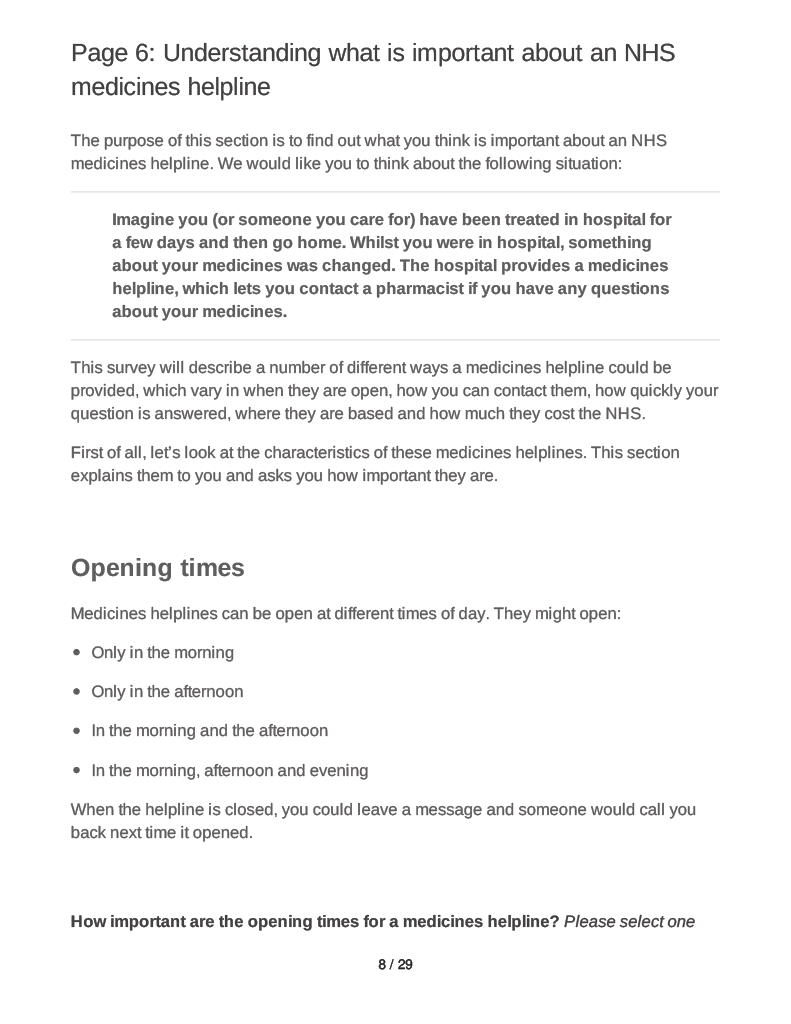
**

**
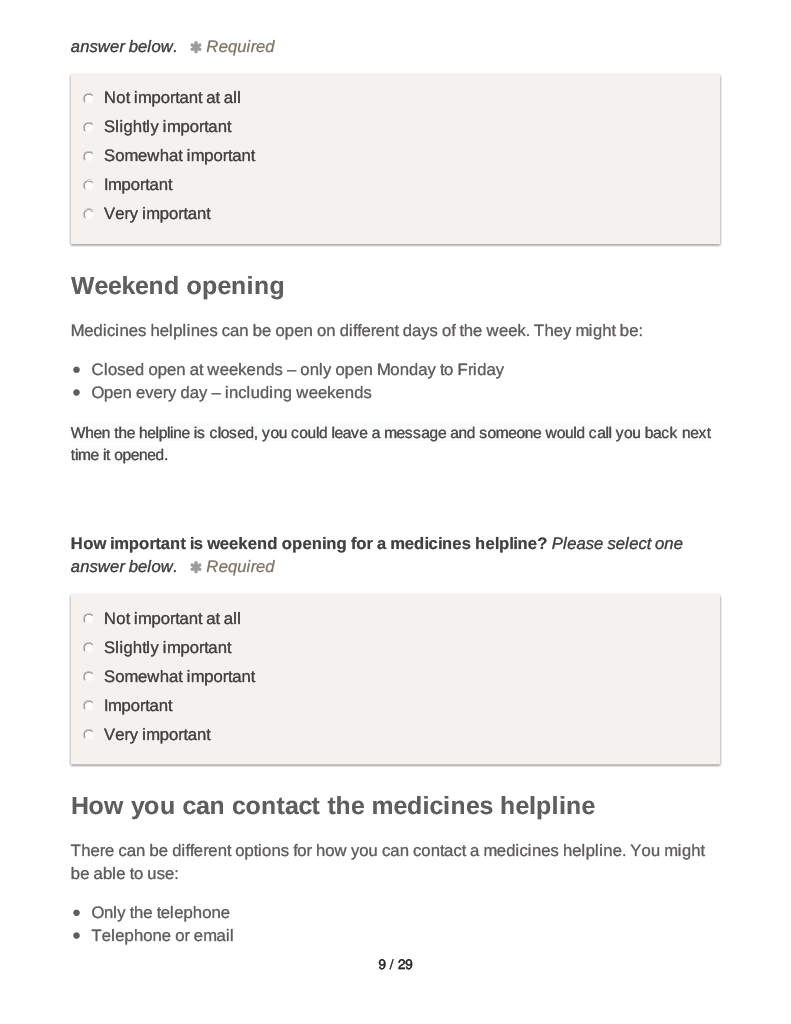
**

**
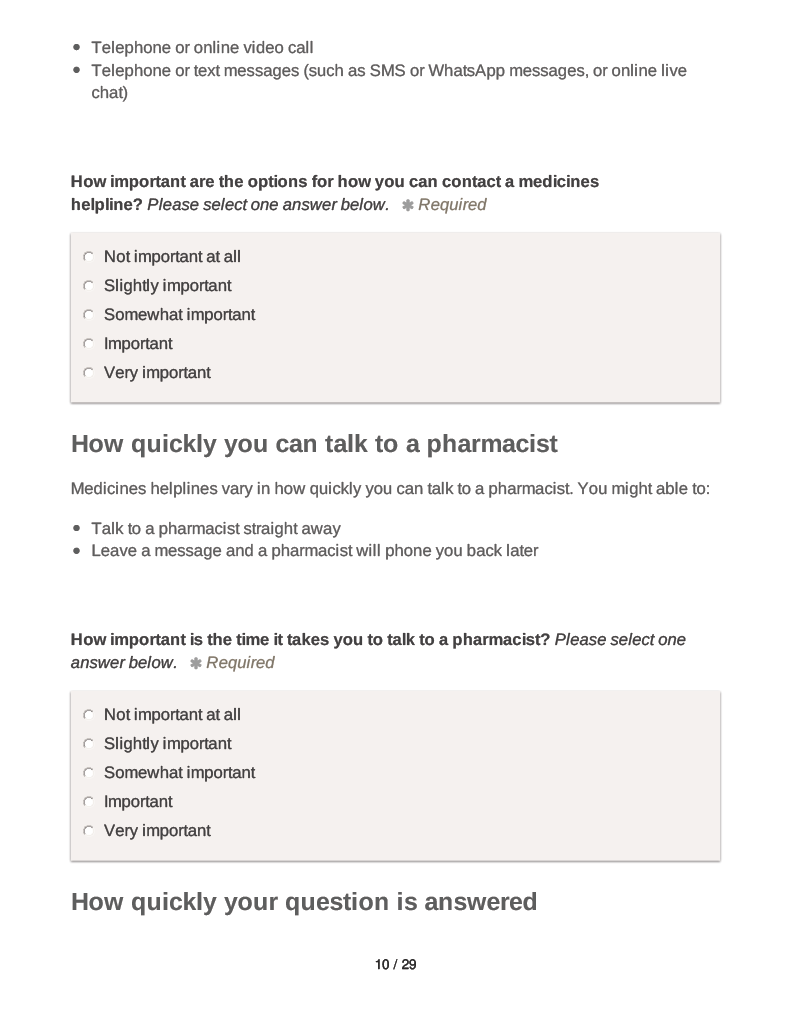
**

**
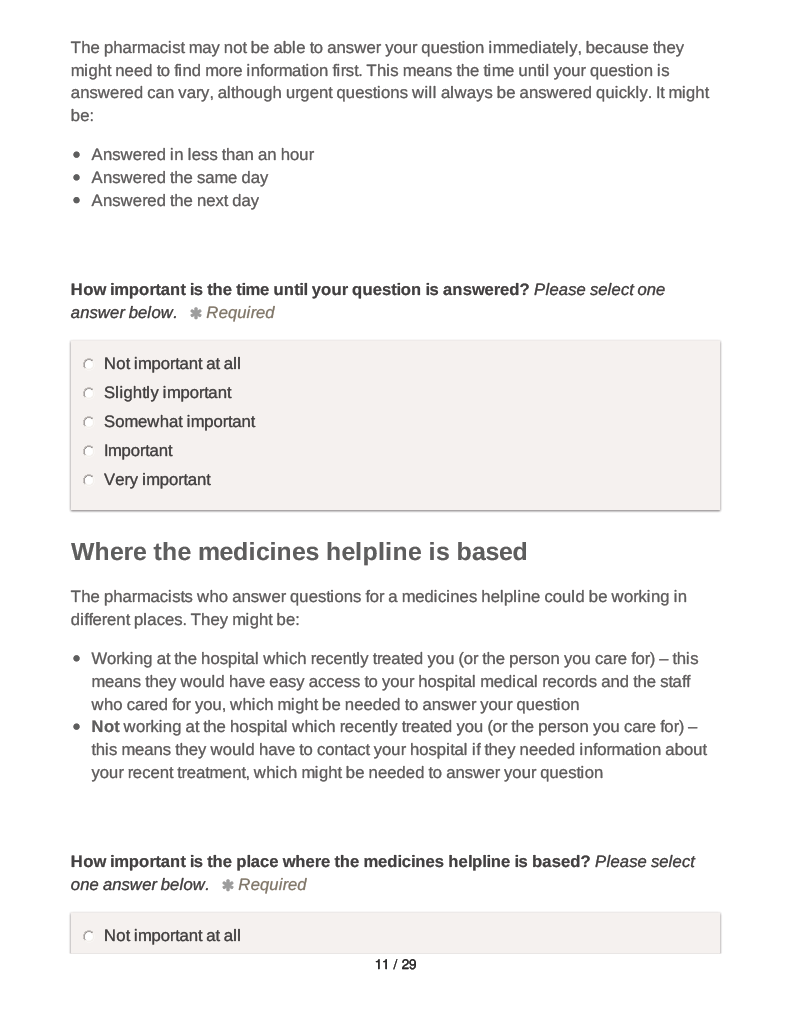
**

**
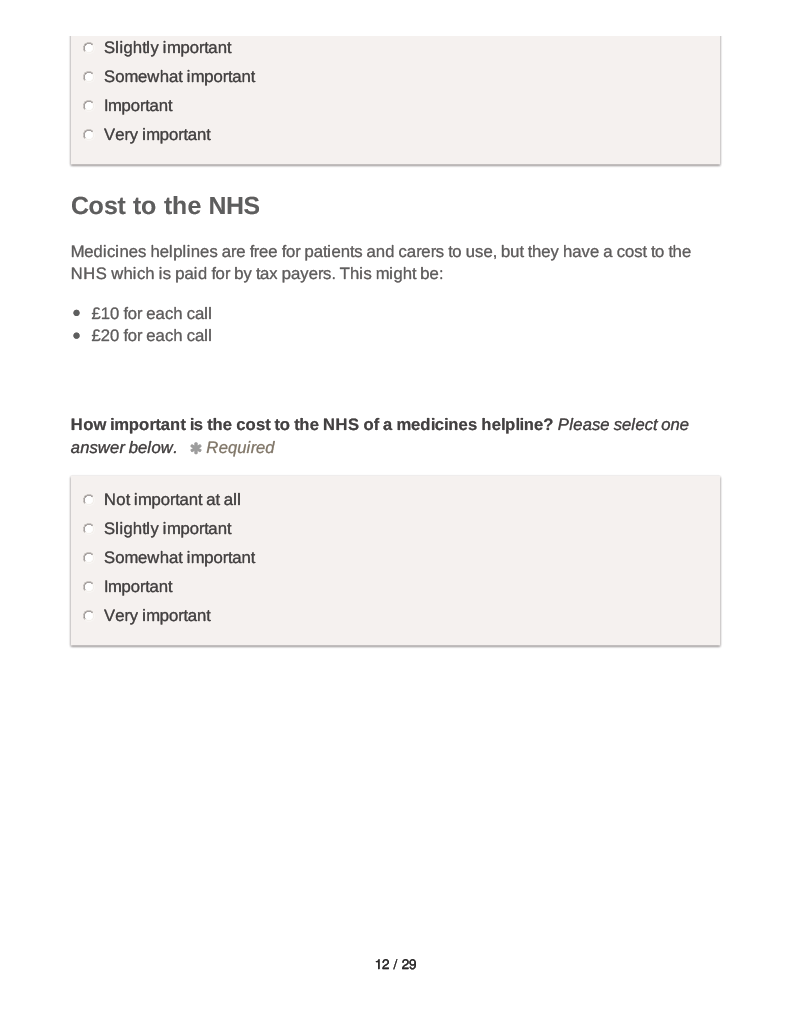
**

**
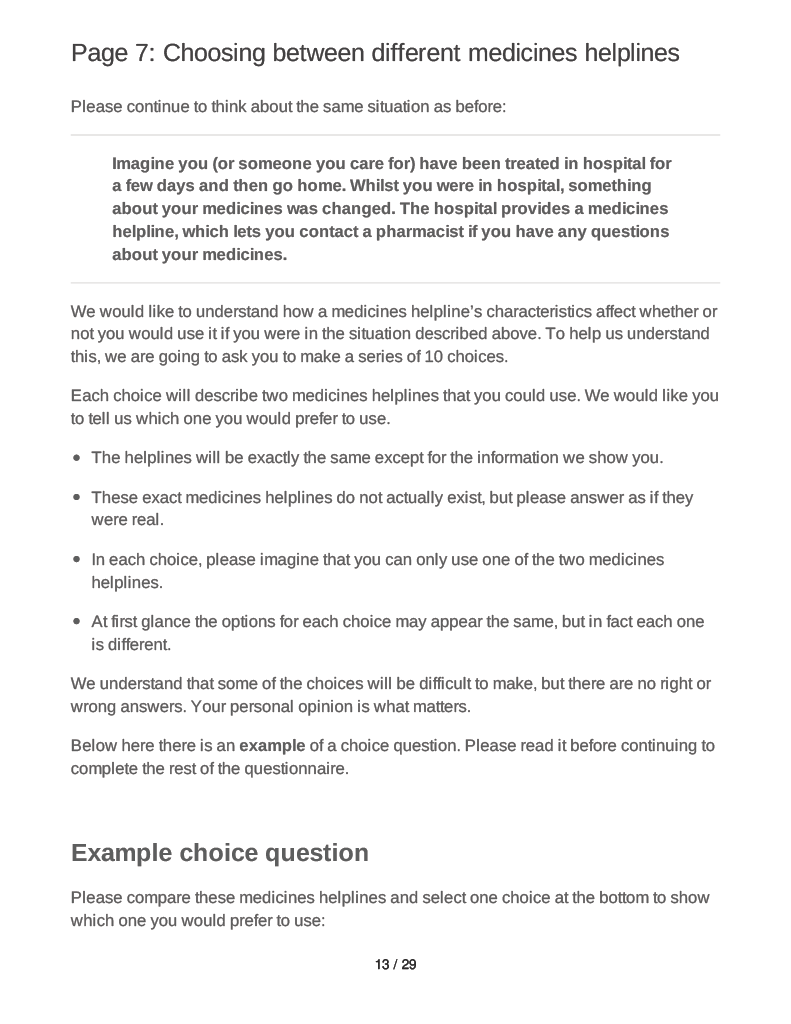
**

**
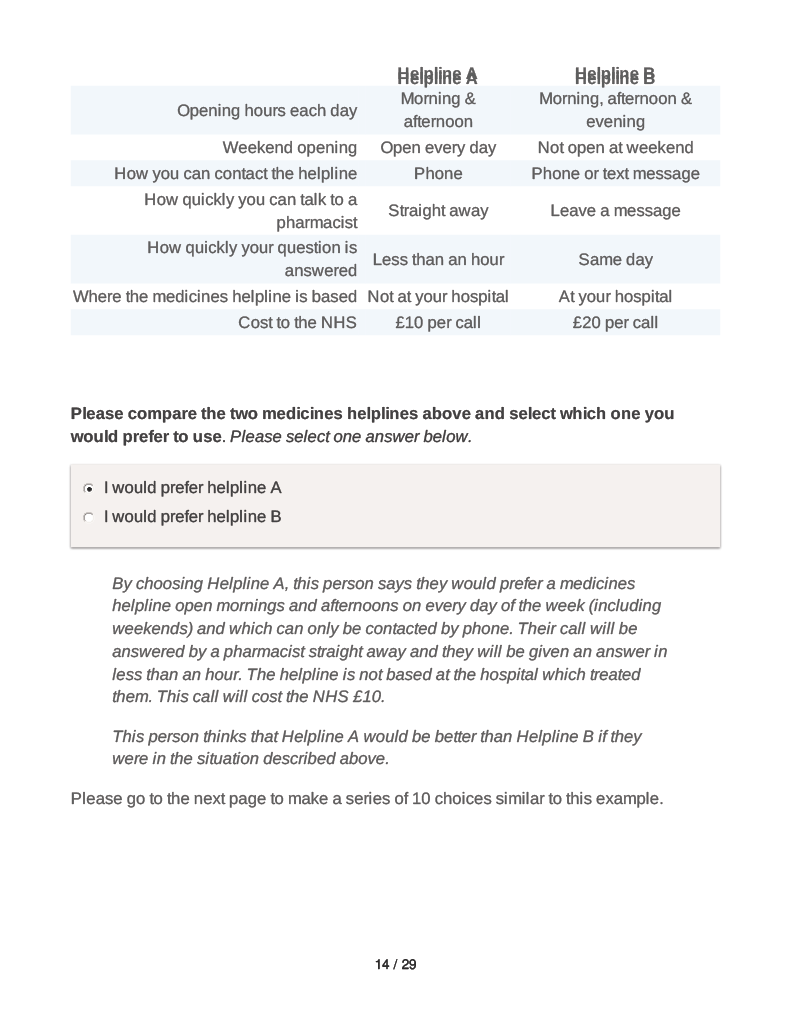
**

**
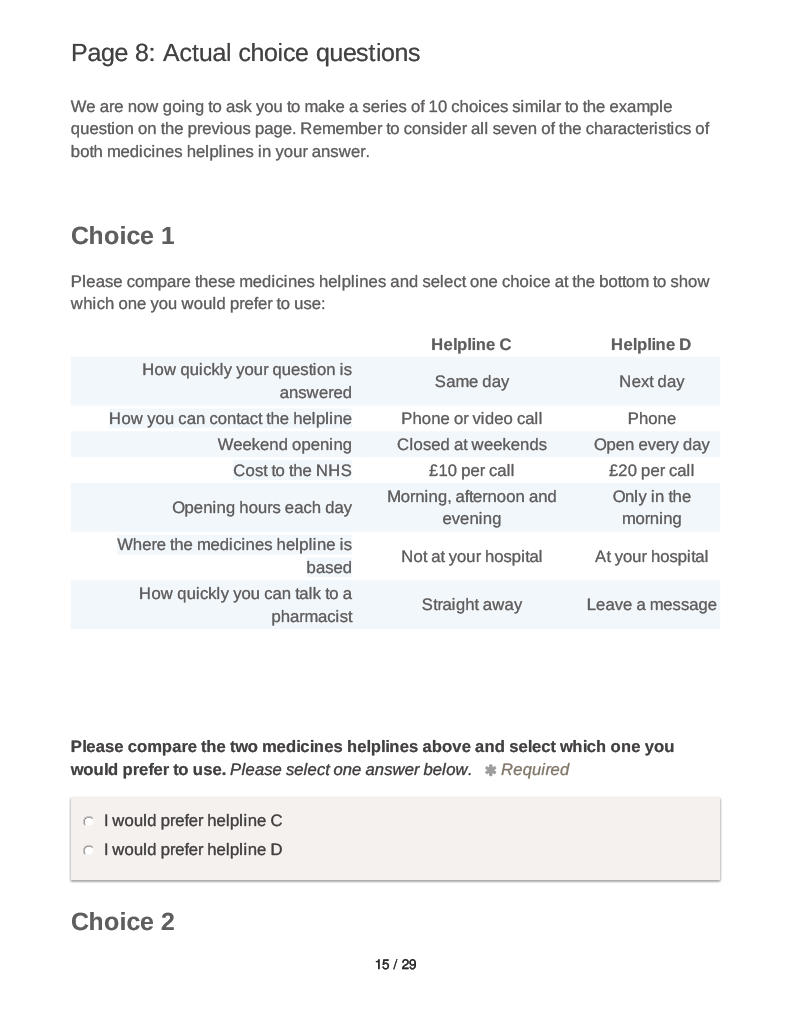
**

**
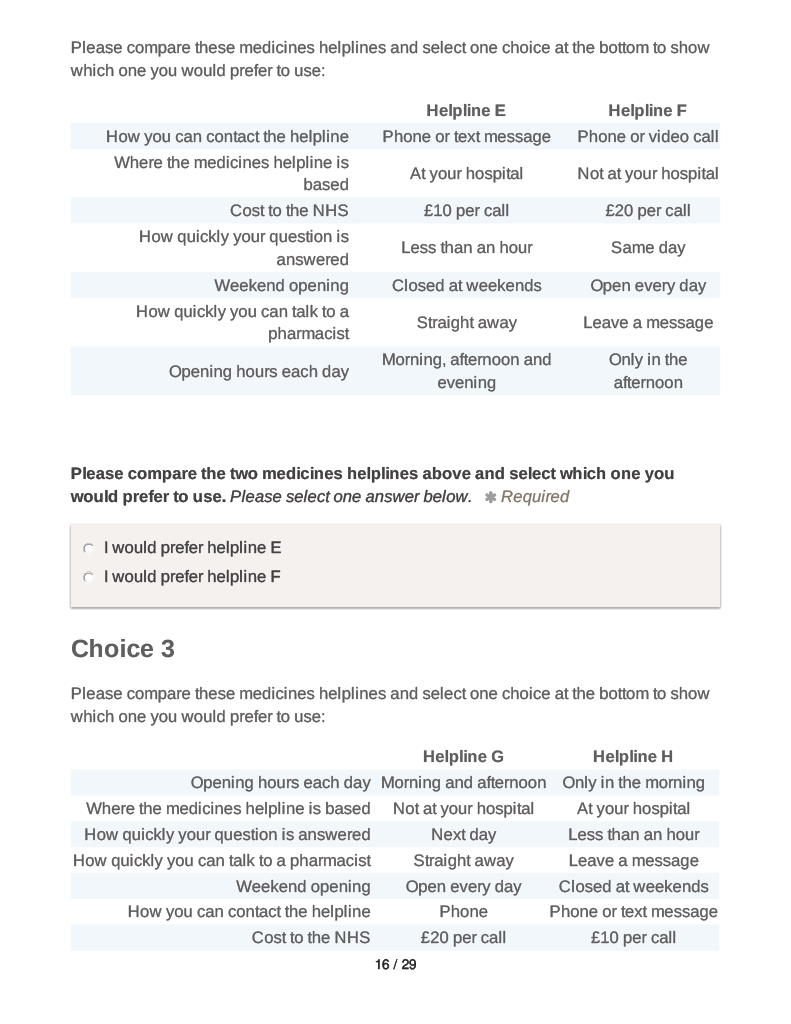
**

**
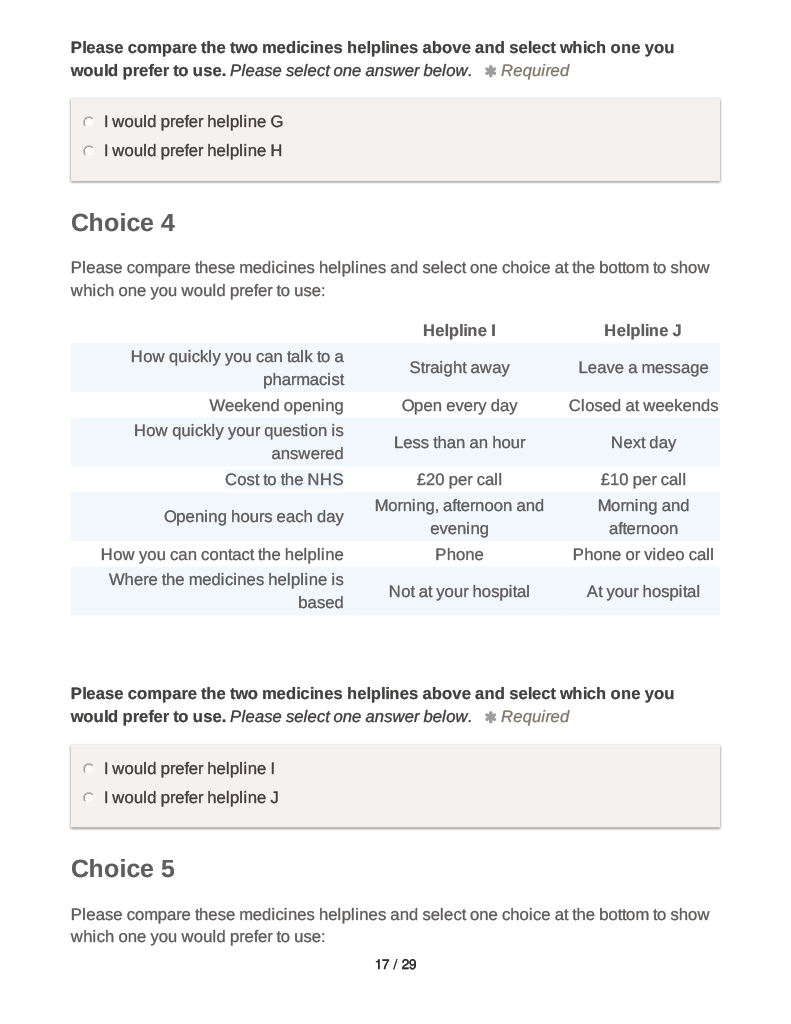
**

**
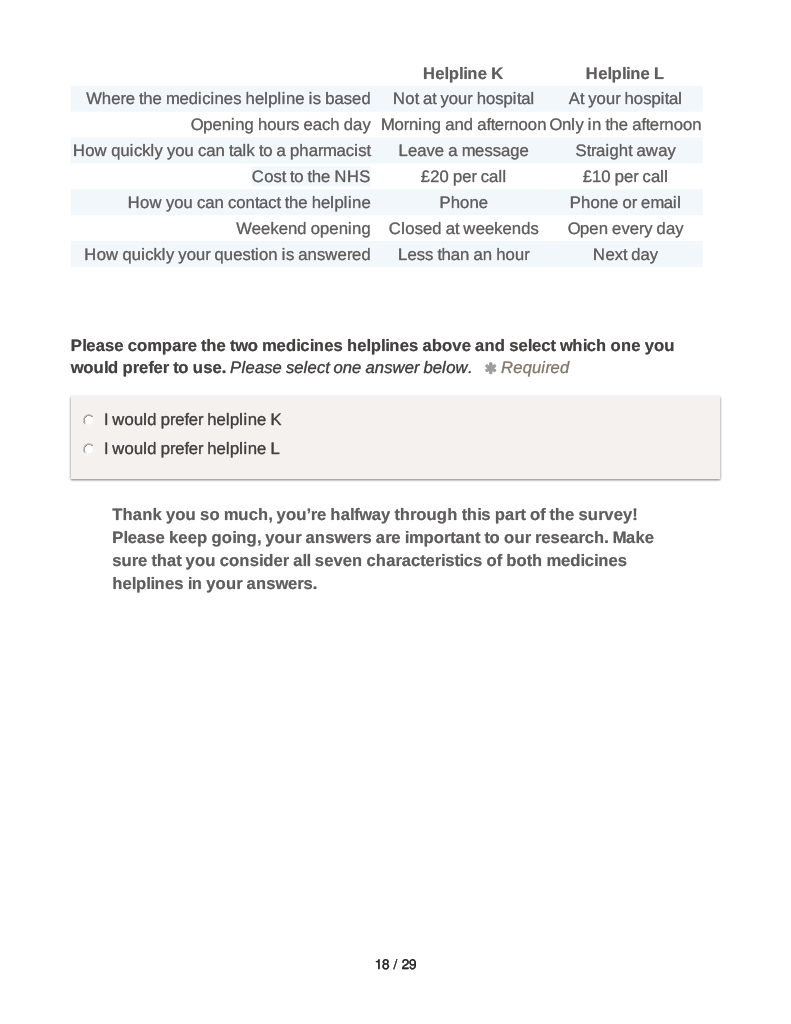
**

**
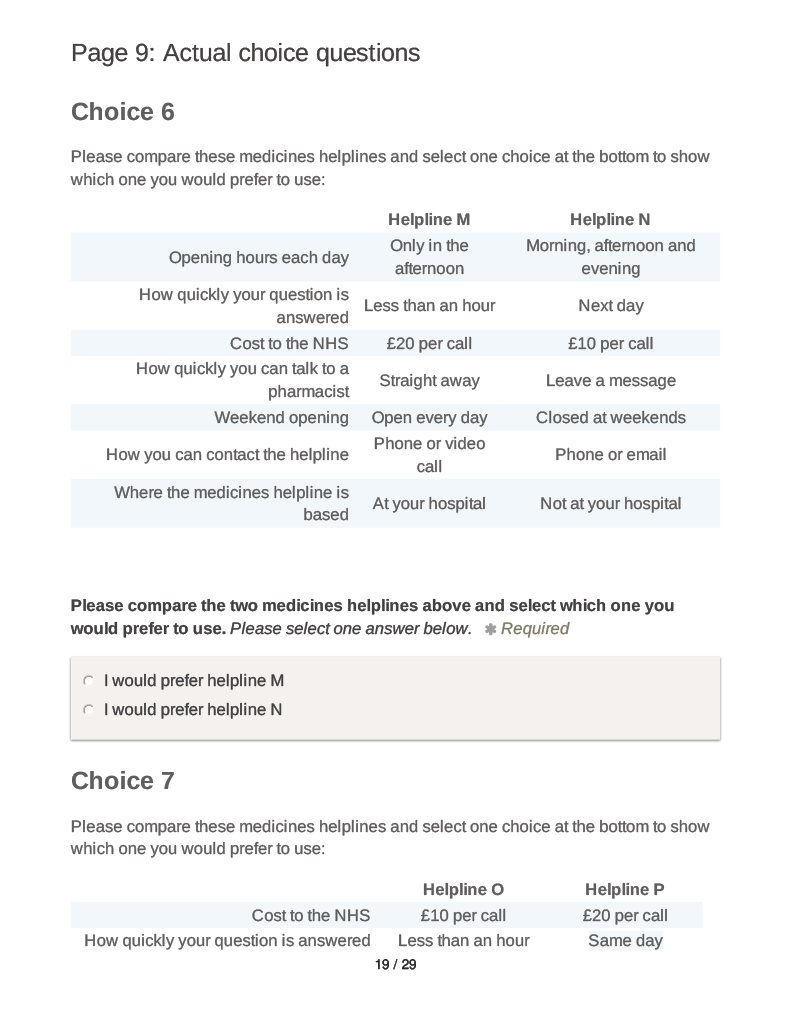
**

**
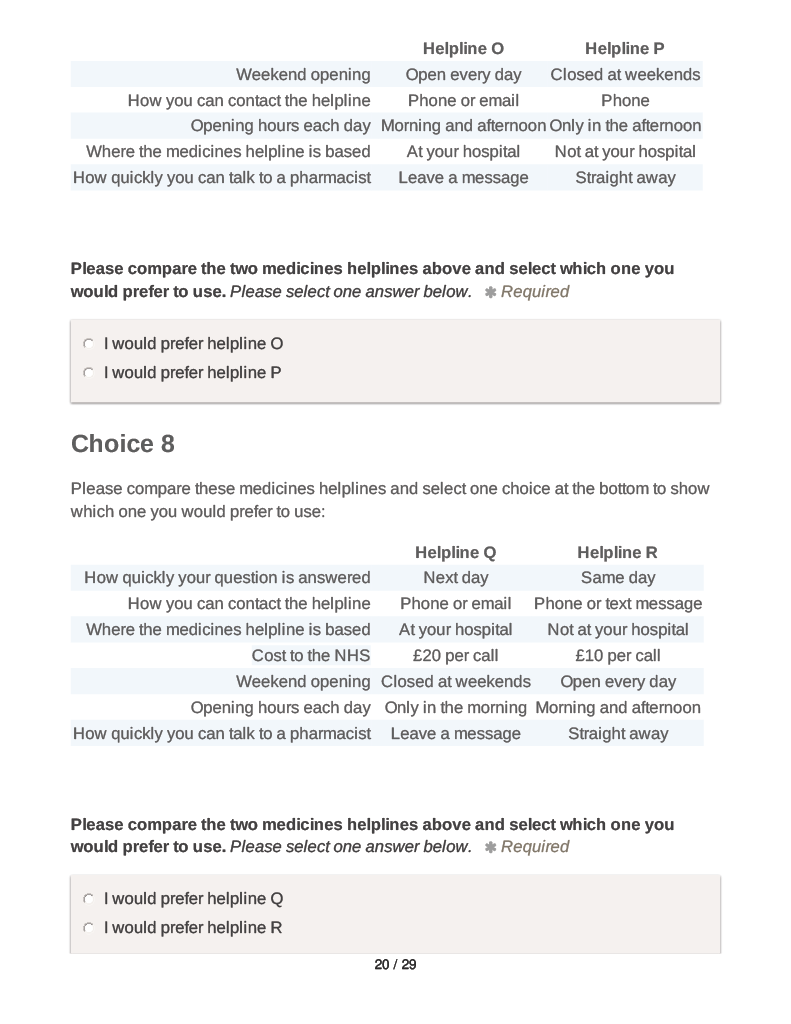
**

**
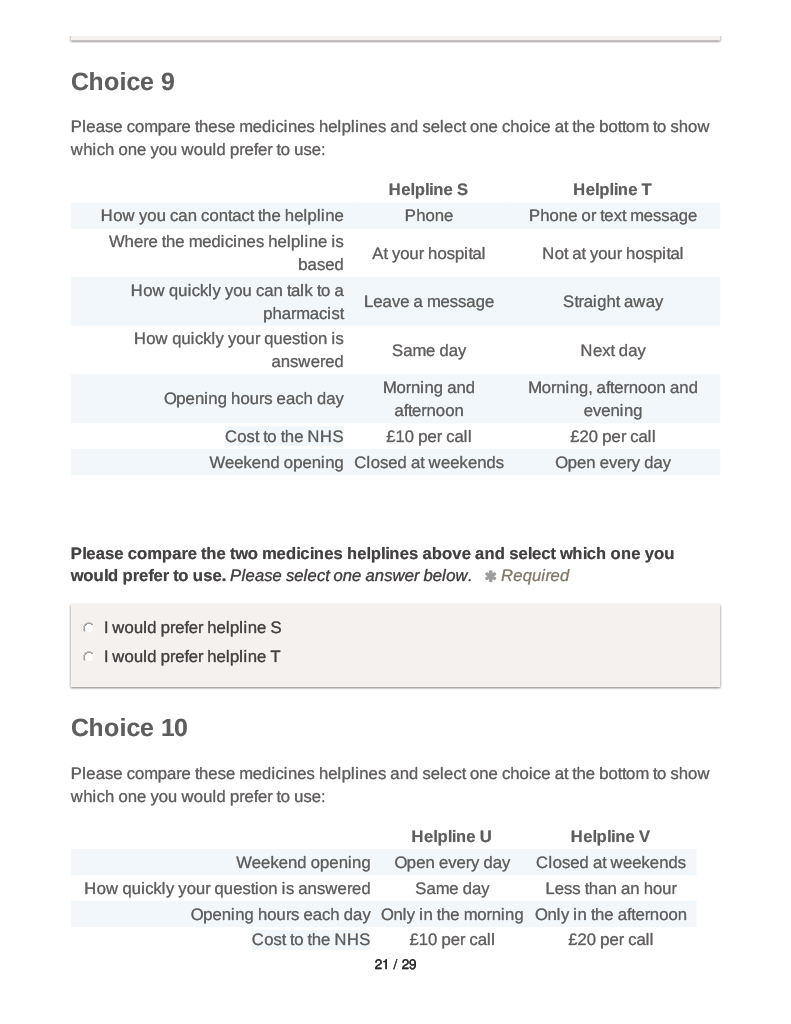
**

**
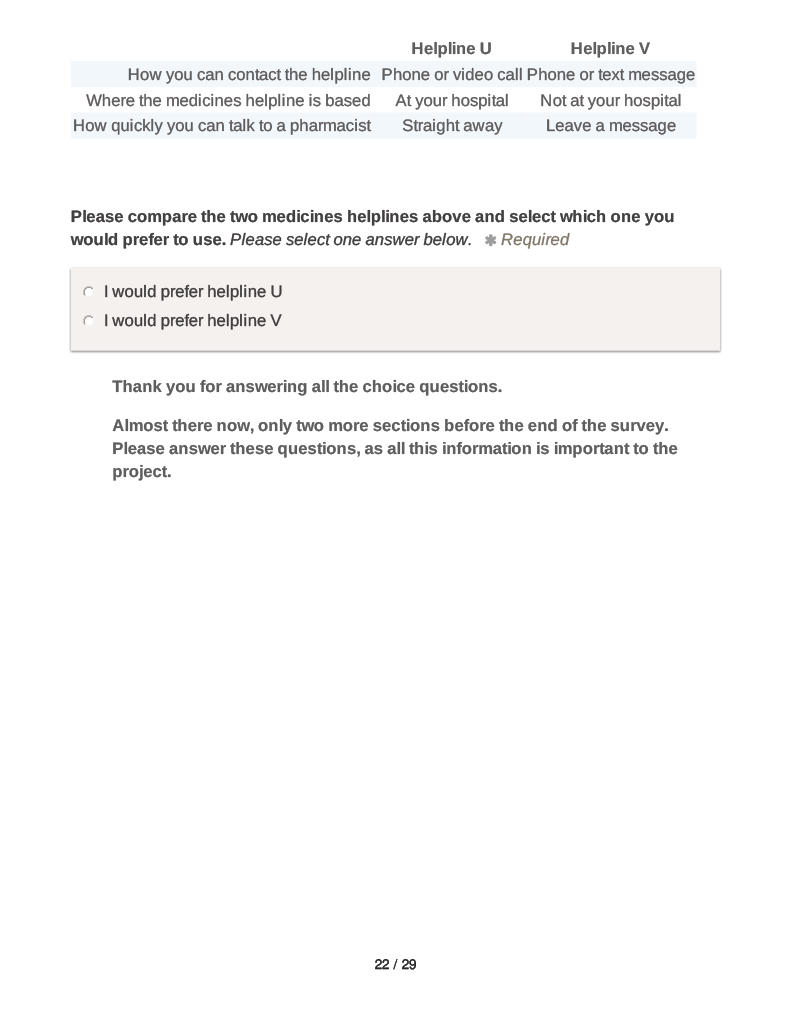
**

**
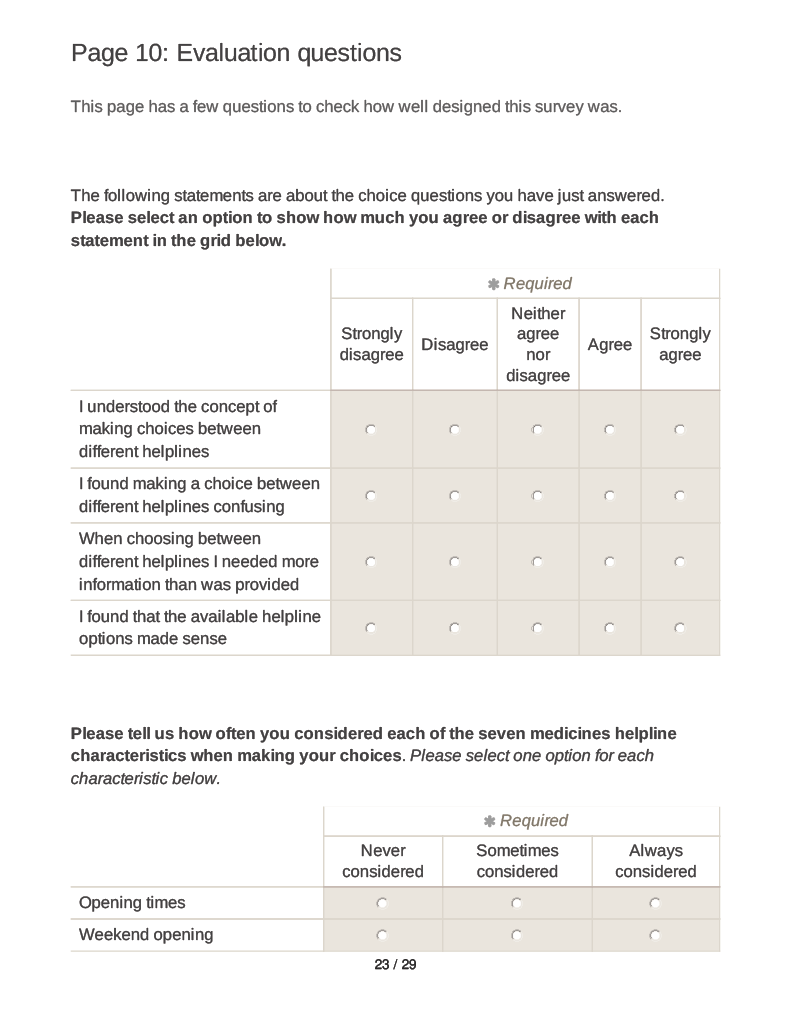
**

**
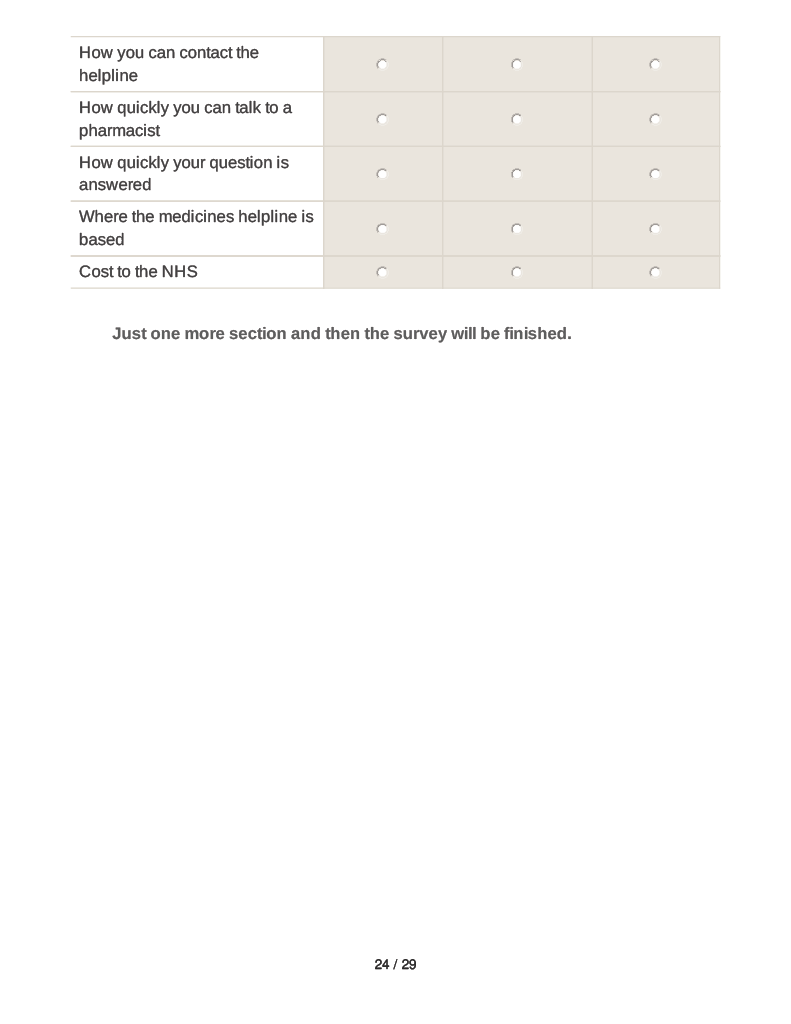
**

**
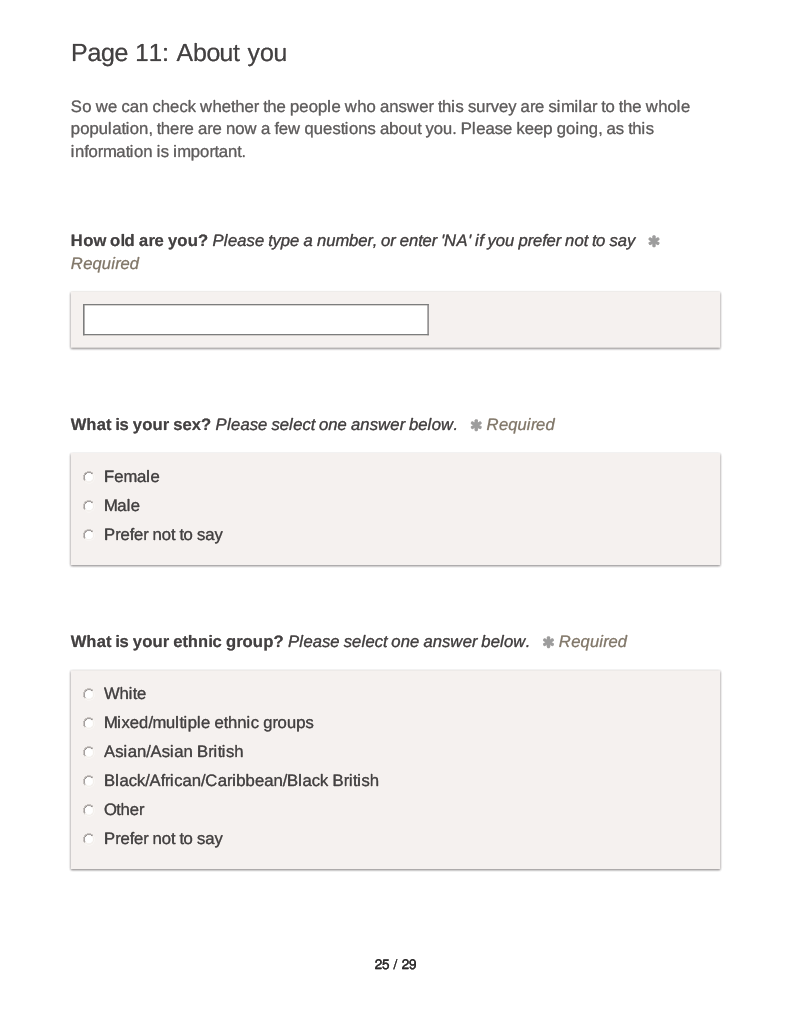
**

**
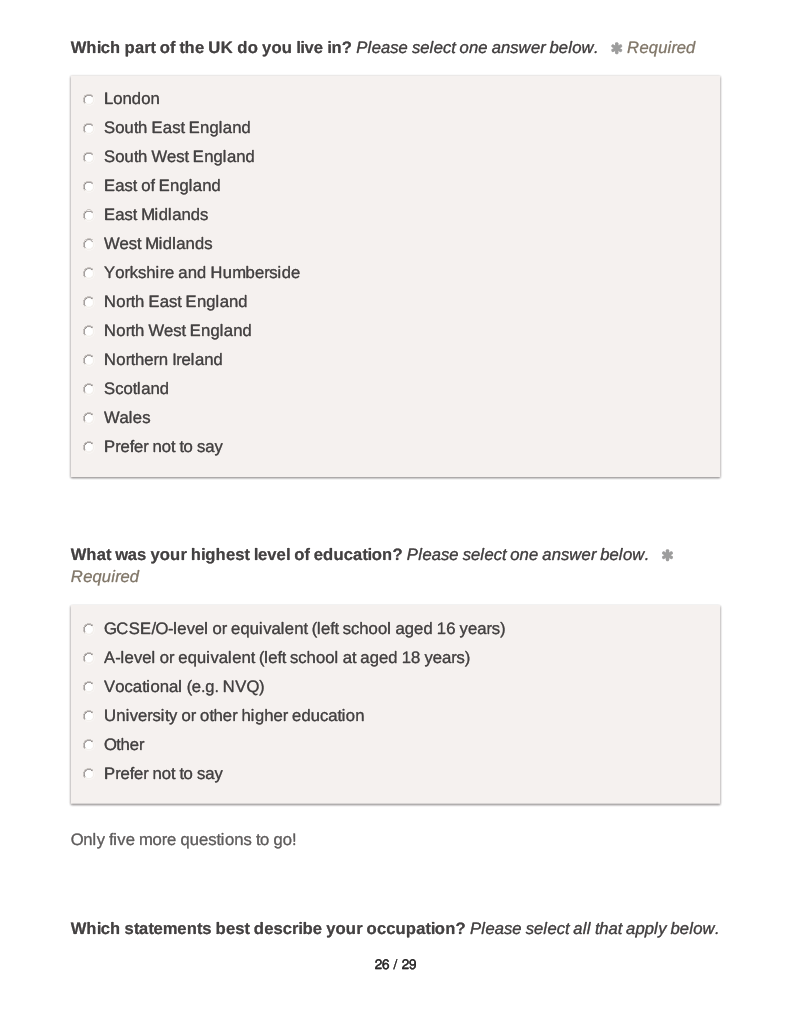
**

**
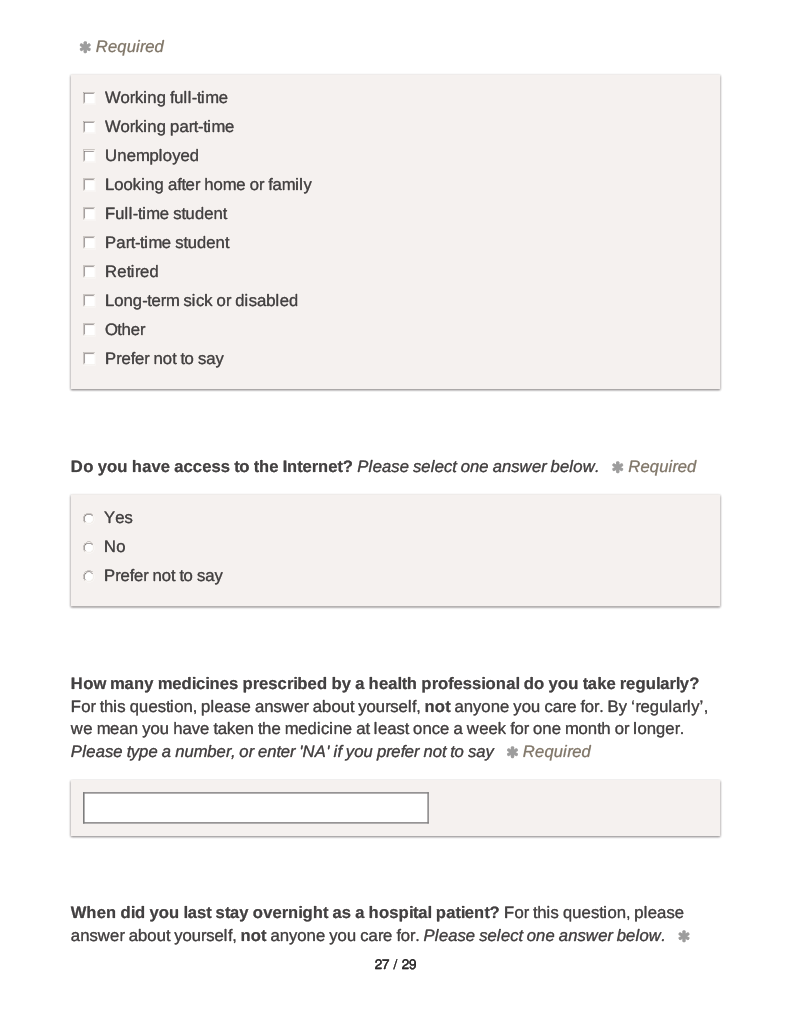
**

**
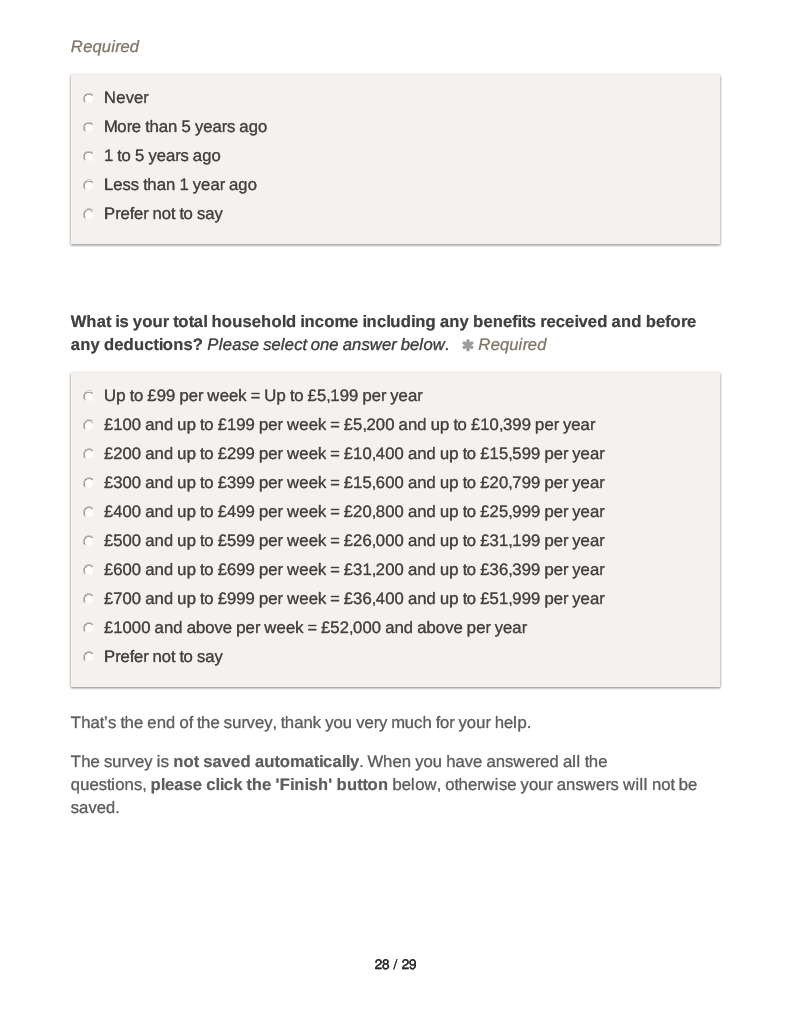
**

**
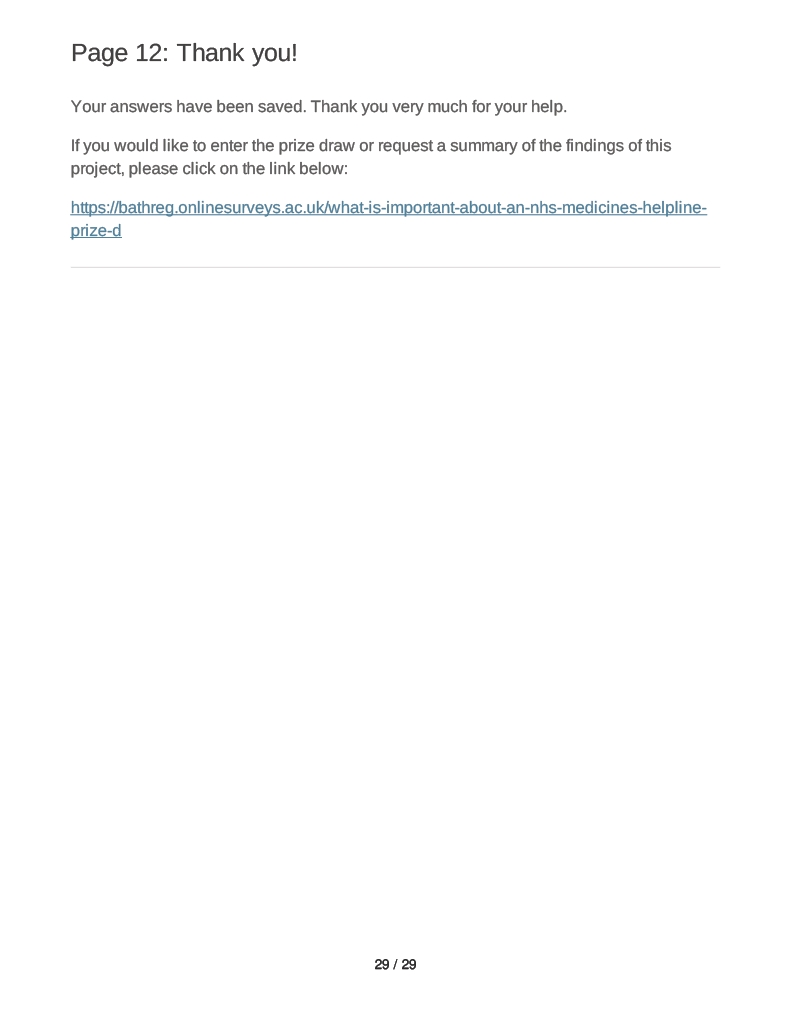
**
